# Impact of a bacterial consortium on the soil bacterial community structure and maize (*Zea mays* L.) cultivation

**DOI:** 10.1038/s41598-021-92517-0

**Published:** 2021-06-22

**Authors:** Laura N. Afanador-Barajas, Yendi E. Navarro-Noya, Marco L. Luna-Guido, Luc Dendooven

**Affiliations:** 1 Laboratory of Soil Ecology, Cinvestav, Mexico City, Mexico; 2 Programa de Biología, Facultad de Ingeniería y Ciencias Básicas, Universidad Central, Bogotá, Colombia; 3 Centro de Investigación en Ciencias Biológicas, Universidad Autónoma de Tlaxcala, Tlaxcala, Mexico

**Keywords:** Biological techniques, Biotechnology, Ecology, Microbiology, Molecular biology, Ecology, Environmental sciences

## Abstract

Microorganisms are often applied as biofertilizer to crops to stimulate plant growth, increase yields and reduce inorganic N application. The survival and proliferation of these allochthonous microorganisms in soil is a necessary requisite for them to promote plant growth. We applied a sterilized or unsterilized not commercialized bacterial consortium mixed with cow manure leachate used by a farmer as biofertilizer to maize (*Zea mays *L.) in a greenhouse experiment, while maize development and the bacterial community structure was determined just before the biofertilizer was applied a first time (day 44), after three applications (day 89) and after six application at the end of the experiment (day 130). Application of sterilized or unsterilized biofertilizer with pH 4.3 and 864 mg NH_4_^+^-N kg^−1^ had no significant effect on maize growth. The application of the biofertilizer dominated by *Lactobacillus* (relative abundance 11.90%) or the sterilized biofertilizer changed the relative abundance of a limited number of bacterial groups, i.e. *Delftia*, *Halomonas*, *Lactobacillus* and *Stenotrophomonas*, without altering significantly the bacterial community structure. Cultivation of maize, however, affected significantly the bacterial community structure, which showed large significant variations over time in the cultivated and uncultivated soil. It was concluded that the bacteria applied as a biofertilizer had only a limited effect on the relative abundance of these groups in uncultivated or soil cultivated with maize.

## Introduction

Plant development is controlled by many factors, such as soil water and nutrient content. Inorganic fertilizer application together with improved plant genotypes has increased crop yields. Increased crop yields, however, requires ever higher application of inorganic fertilizer, mostly nitrogen, phosphorus and potassium, which can negatively affect the environment. Excess application of inorganic N for instance, can lead to acidification of soil or eutrophication of rivers or aquifers as nitrate the end product of the nitrification process is easily leached out^1^. Inorganic N in soil contributes also to the emission of nitrous oxide (N_2_O), a potent greenhouse gas. Ammonium (NH_4_^+^) is oxidized in soil under aerobic conditions to nitrite (NO_2_^−^) and nitrate (NO_3_^−^), i.e. nitrification, whereby N_2_O is produced and under anaerobic conditions NO_3_^−^ can be reduced to N_2_O and or dinitrogen (N_2_)^2^.

Although the application of inorganic fertilizer has increased crop yields, environmental concerns have led to the search of methods that might help to reduce its use, while maintaining or even improving crop yields. Vertical and organic farming, precision agriculture or applying fertilizer only when the crop requires it has helped to reduce inorganic fertilizer application rates. While these management strategies are helpful in reducing the need of inorganic fertilizer, additional solutions are still needed for growers who do not have the means to perform vertical, organic or precision farming^3,4^. One promising solution that has not fully been explored for cereal production is the use of a bioconsortium as biofertilizers^5,6^.

Plant growth promoting microorganism (PGPM) are often applied as biofertilizers to seeds, roots, plant or soil. They promote plant growth by supplying essential nutrients, such as nitrogen and phosphorus^7^. Lactic acid bacteria (LAB) have been used for decades in agricultural practices with a promising potential as PGPMs. They have been applied as biofertilizer, or to control diseases, improve soil fertility and promote plant growth^8^. Lactobacilli can stimulate plant growth as they produce auxins, volatile fatty acids and plantericin. They have also been applied to soil against phytopathogenic bacteria and as biocontrol agents for their antifungal activity^8,9^.

The question remains, however, how allochthonous microorganisms, i.e. introduced into the soil, will survive and stimulate plant growth^10^. The soil environment is highly competitive. Microorganisms applied to soil might not be physically protected against predation^11–13^ and it might be that a positive effect is the result of nutrients released after their predation. Additionally, the movement of microorganisms is limited in soil so it might be difficult for them to get in contact with the growing roots when they are applied on the soil surface. In a previous research, we applied a single strain of *Bacillus subtilis* with plant growth promoting capacities, but it did not accelerate plant development^14^. Therefore, in this study, a consortium dominated by lactobacilli mixed with cow manure leachate and applied by a local farmer as biofertilizer to increase maize yields was used. The lactobacilli were mixed with the cow manure leachate to provide the microorganisms with nutrients and as an additional source of nutrients for the cultivated crops. The sterilized and unsterilized consortium mixed with cow manure leachate was applied to maize plants cultivated in the greenhouse, while crop development and the bacterial community in the bulk and rhizosphere soil were monitored for 130 days. The bacterial community was also monitored in the uncultured soil, which served as a control. We hypothesized that application of the biofertilizer would change the bacterial community structure and stimulate maize growth. As such, the objective of this study was to study the effect of the application of a biofertilizer on plant growth and how it altered the bacterial community structure in uncultivated soil and cultivated with maize.

## Results

### Characterization of the biofertilizer, soil and maize plant

The pH of the biofertilizer was acidic (4.3) with an electrolytic conductivity (EC) of 20.4 dS m^−1^ and the NH_4_^+^ content was 864 mg kg^−1^ (Supplementary Table S1 online). The application of sterilized or unsterilized biofertilizer did not alter the soil pH, but cultivation of maize increased it significantly (F = 20.27, *P* < 0.001) (Supplementary Tables S2, S3, S4 online). The concentration of NH_4_^+^ and NO_2_^−^ was not affected significantly by cultivation of maize or the application of biofertilizer or the sterilized biofertilizer. The concentration of NO_3_^−^ was affected highly significantly by cultivation of maize (F = 47.55, *P* < 0.001), but application of biofertilizer or sterilized biofertilizer had no effect on it. The concentration of NO_3_^−^ increased significantly in the uncultivated soil over time (*P* < 0.05), but not in the maize cultivated soil. None of the maize characteristics was affected significantly by treatment, but most of them increased highly significantly over time (*P* < 0.001) (Table 1).

**Table 1:** Characteristics of maize plants (*Zea mays* L.) in soil left unamended, amended with biofertilizer or sterilized biofertilizer after 44, 89 and 130 days.

	Root length (cm)					Plant height (cm)				
	Sampling days					Sampling days				
Treatment	44	89	130	F value	*P* value	44	89	130	F value	*P* value
Unamended	27.7^a^ A^b^ b^c^	78.0 A a	82.9 A a	81.56	< 0.001	48.6 A c	99.1 A b	140.7 A a	133.39	< 0.001
Biofertilizer	24.1 A b	79.0 A a	80.2 A a	36.97	0.003	38.2 A c	100.8 A b	136.9 A a	74.54	< 0.001
Sterilized biofertilizer	46.5 A b	85.6 A a	94.2 A a	15.10	0.015	52.2 A c	98.1 A b	149.7 A a	340.96	< 0.001
F value	4.95	0.76	0.58			1.78	0.06	4.43		
*P* value	0.094	0.528	0.603			0.285	0.943	0.130		

	Root fresh weight (g)					Root dry weight (g)				
Unamended	3.7 A b	32.1 A a	30.6 A a	386.61	< 0.001	1.9 A c	6.2 A b	30.9 A a	30.67	0.014
Biofertilizer	2.2 A a	37.3 A a	37.6 A a	19.84	0.050	1.8 A b	7.0 A b	29.1 A a	143.23	0.002
Sterilized biofertilizer	7.6 A b	51.2 A a	31.8 A a	24.84	0.008	2.8 A b	10.2 A b	34.6 A a	31.08	0.011
F value	2.90	0.88	0.50			1.16	1.20	0.80		
*P* value	0.185	0.489	0.654			0.409	0.406	0.521		

	Shoot fresh weight (g)					Shoot dry weight (g)				
Unamended	15.7 A b	139.5 A a	145.1 A a	213.42	< 0.001	3.8 A c	7.8 A b	23.2 A a	60.68	0.002
Biofertilizer	10.7 A b	131.4 A a	148.5 A a	223.41	< 0.001	3.0 A c	9.4 A b	27.2 A a	1656.65	< 0.001
Sterilized biofertilizer	32.6 A b	142.5 A a	142.5 A a	34.36	0.004	4.4 A a	8.8 A a	25.4 A a	7.56	0.054
F value	2.50	0.15	1.70			2.76	0.87	1.54		
*P* value	0.214	0.864	0.851			0.186	0.498	0.356		

^a^Mean of three samples, ^b^values with the same capital letter are not significantly different between the treatments, i.e. within the columns, ^c^values with the same letter are not significantly different over time, i.e. within the rows. A non-parametric test was used, i.e. t1way test of the WRS2 package, to test the effect of time and treatment (a collection of robust statistical methods^51^).

### Characteristics of the biofertilizer and its bacterial community structure

**Figure 1:**
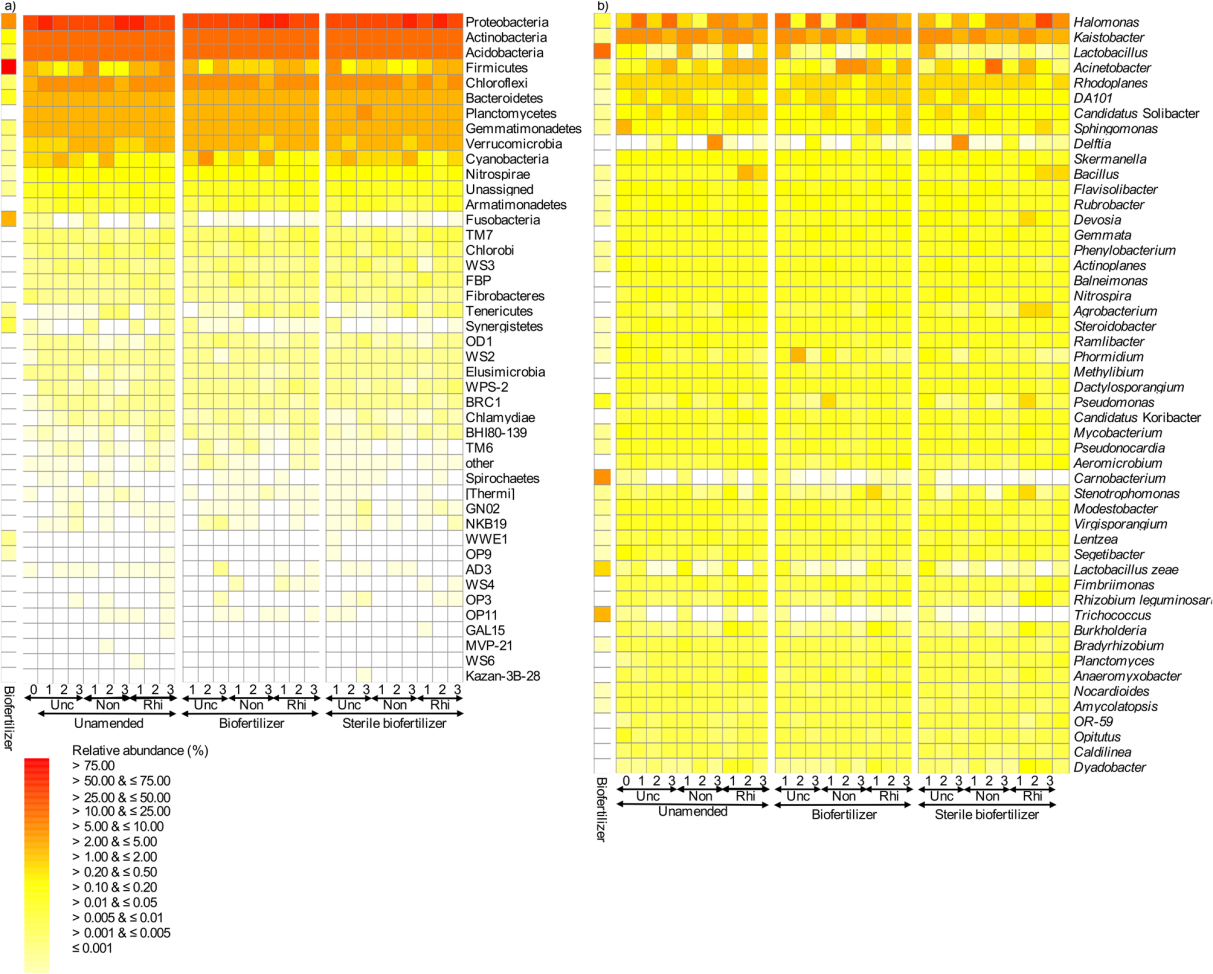
Heatmap with the relative abundance of (**a**) the bacterial phyla and (**b**) the 50 most abundant bacterial genera in the uncultivated (Unc), non-rhizosphere (Non) and rhizosphere soil (Rhi) left unamended soil (Unamended) or amended with biofertilizer (Biofertilizer) or the sterile biofertilizer (Sterile biofertilizer) at the onset of the experiment (0), and after 44 days (1), 89 days (2) and 144 days (3).

Overall, 21,282 sequences were obtained from the biofertilizer sample that represented 553 OTUs. The OTUs detected in the biofertilizer belonged to 21 bacterial phyla and 91 genera (Fig. 1). The bacterial community in the biofertilizer was dominated by Firmicutes (relative abundance 91.33 ± 4.99%), mostly *Lactobacillus* (relative abundance 11.90 ± 2.40%) (Fig. 1).

### Rarefication curves and alpha diversity of the bacterial community in soil

Overall, 2,570,199 16S rRNA sequences were analysed, which yielded 36,686 OTUs. The rarefication curve of the number of sequences versus the number of OTUs was asymptotic so the analysis of more sequences would yield only a limited number of new OTUs (Supplementary Fig. S1).

Application of biofertilizer had no significant effect on the alpha diversity indices after 89 or 130 days (Supplementary Table S5, Fig. S2 online). Cultivation of maize plants had mostly a highly significant effect on the alpha diversity indexes after 44 and 130 days, but not after 89 days.

### Effect of application of biofertilizer or sterile biofertilizer on the bacterial community

The bacterial community structure in the soil at the onset of the experiment was dominated by Proteobacteria (47.68 ± 4.30%) and Actinobacteria (20.63 ± 1.33%), mostly *Kaistobacter* (5.66 ± 1.68%) and *Sphingomonas* (2.43 ± 1.06%) (Fig. 1). After 130 days, the largest increases in the relative abundance in the uncultivated unamended soil were found for members of *Acinetobacter*, *Halomonas*, Nitrospirae, OD1 and WS2 and the largest decreases in *Lactobacillus*, *Pseudomonas*, *Phormidium*, *Sphingomonas* and TM7. The enrichment of members of *Halomonas* (11.18 ± 6.52%) in the unamended uncultivated was such that it became the dominant bacterial genera after 130 days.

**Figure 2:**
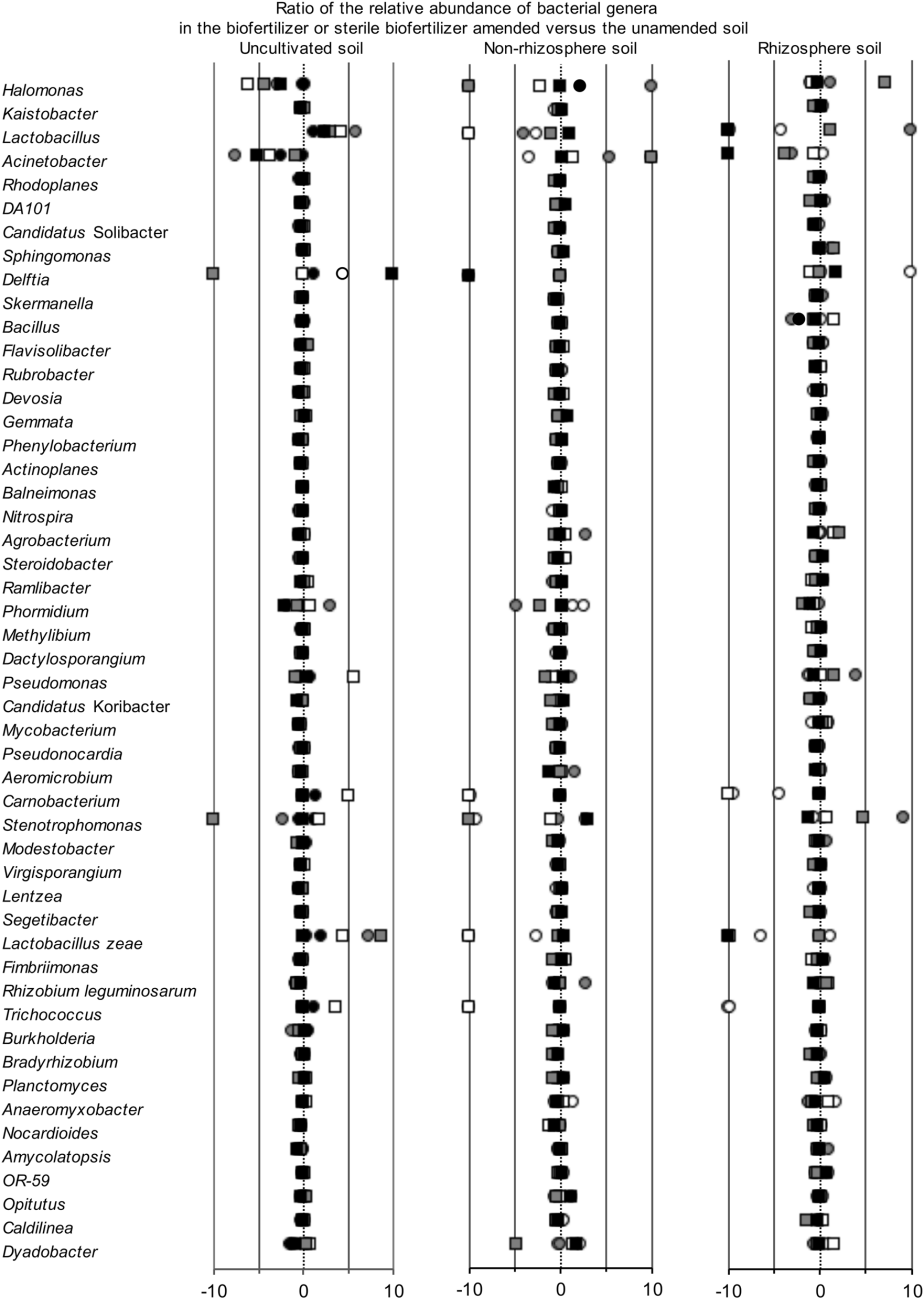
Ratio of the relative abundance of the 50 most abundant bacterial genera in the biofertilizer amended soil after 44 days (white circle), 89 days (grey circle) and 130 days (black circle) or amended with sterile biofertilizer after 44 days (white square), 89 days (grey square) and 130 days (black square) versus the unamended (**a**) uncultivated soil, (**b**) non-rhizosphere soil and (**c**) rhizosphere soil.

Application of biofertilizer or sterile biofertilizer had a small effect on most bacterial groups or OTUs, although a limited number were affected strongly, e.g. *Delftia*, *Halomonas*, *Lactobacillus* and *Stenotrophomonas* (Fig. 2). The PCA and PCoA (Data not shown) showed little effect of application of biofertilizer on the bacterial community independent of the taxonomic level considered, but visualized changes in the composition of the bacterial community structure in the uncultivated soil over time most accentuated when considering all bacterial groups assigned to the level of genus (Supplementary Figs. S2, S3, S4 online). Consequently, the perMANOVA analysis indicated that application of biofertilizer had no significant effect on the bacterial community structure, but time had a highly significant effect considering the bacterial phyla and genera (*P* ≤ 0.002).

**Figure 3:**
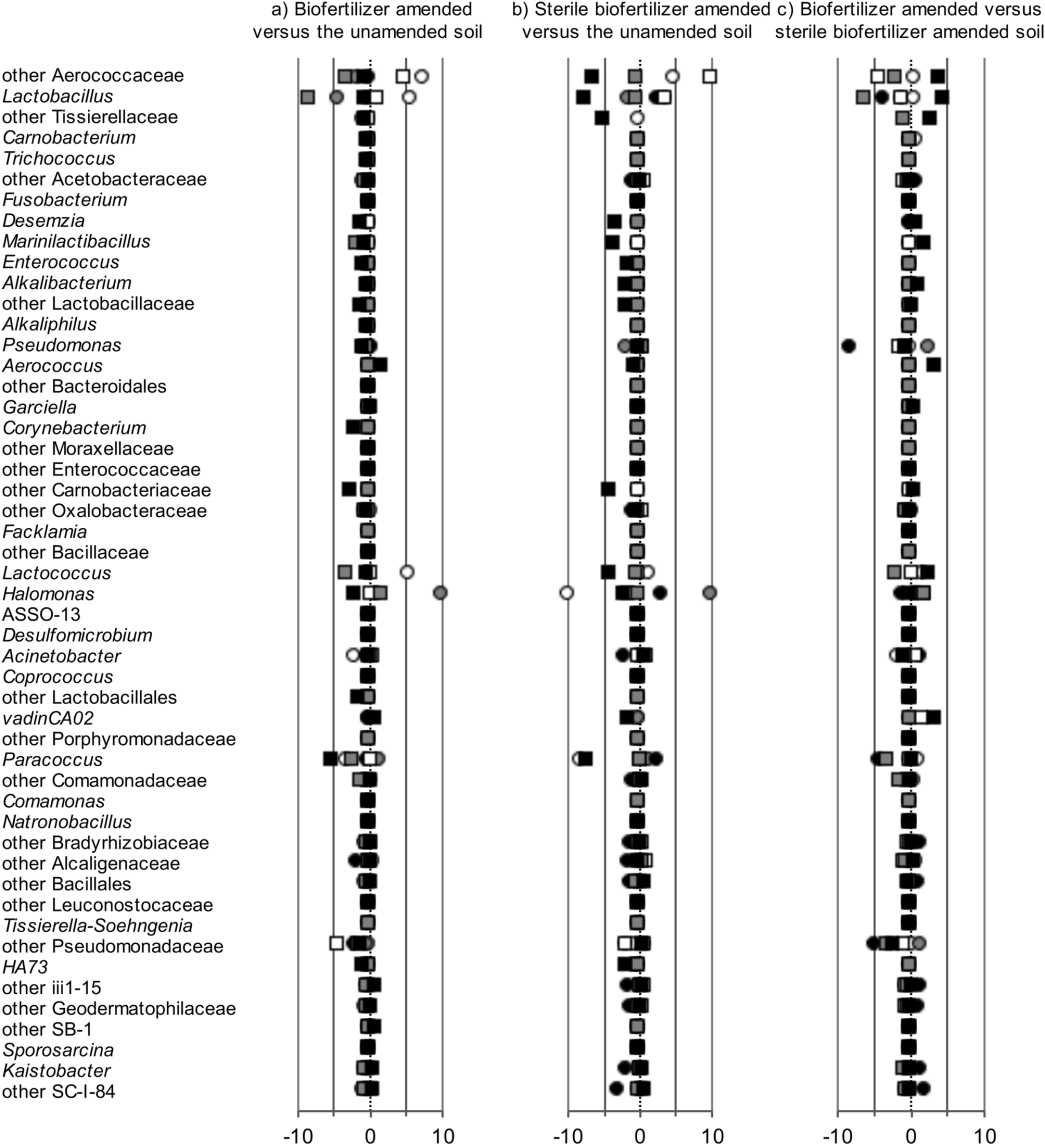
Ratio of the relative abundance of the 50 most abundant bacterial groups assigned up to the taxonomic level of genus applied with the biofertilizer in (**a**) the biofertilizer versus the unamended soil and (**b**) sterile biofertilizer amended versus the unamended soil and (**c**) the ratio in the biofertilizer versus the sterile biofertilizer amended uncultivated soil (white circle), non-rhizosphere soil (grey circle) and rhizosphere soil (black circle) after 89 days or the uncultivated soil (white square), non-rhizosphere soil (grey square) and rhizosphere soil (black square) after 130 days.

Only a limited number of the bacterial groups applied with the biofertilizer affected the relative abundance of these groups in soil considering those assigned up to the level of genus (Fig. 3, Supplementary Table S6 online) or considering the different OTUs (Supplementary Table S7 online). The effect of the bacterial groups applied with the biofertilizer on these groups in soil, however, often changed over time, was mostly negative instead of positive, and was different in the uncultivated, non-rhizosphere and rhizosphere soil (Fig. 3a). Additionally, the application of sterile biofertilizer had often an effect on the same bacterial groups as when the biofertilizer was applied (Fig. 3b). Consequently, only a few bacterial groups applied with the biofertilizer altered the relative abundance of these groups in soil as compared to those affected when the sterile biofertilizer was applied to soil (Fig. 3c). And once again, the effect often changed over time, was often negative instead of positive, and was different in the uncultivated, non-rhizosphere and rhizosphere soil.

### Effect of cultivation of maize on the bacterial community structure

**Figure 4:**
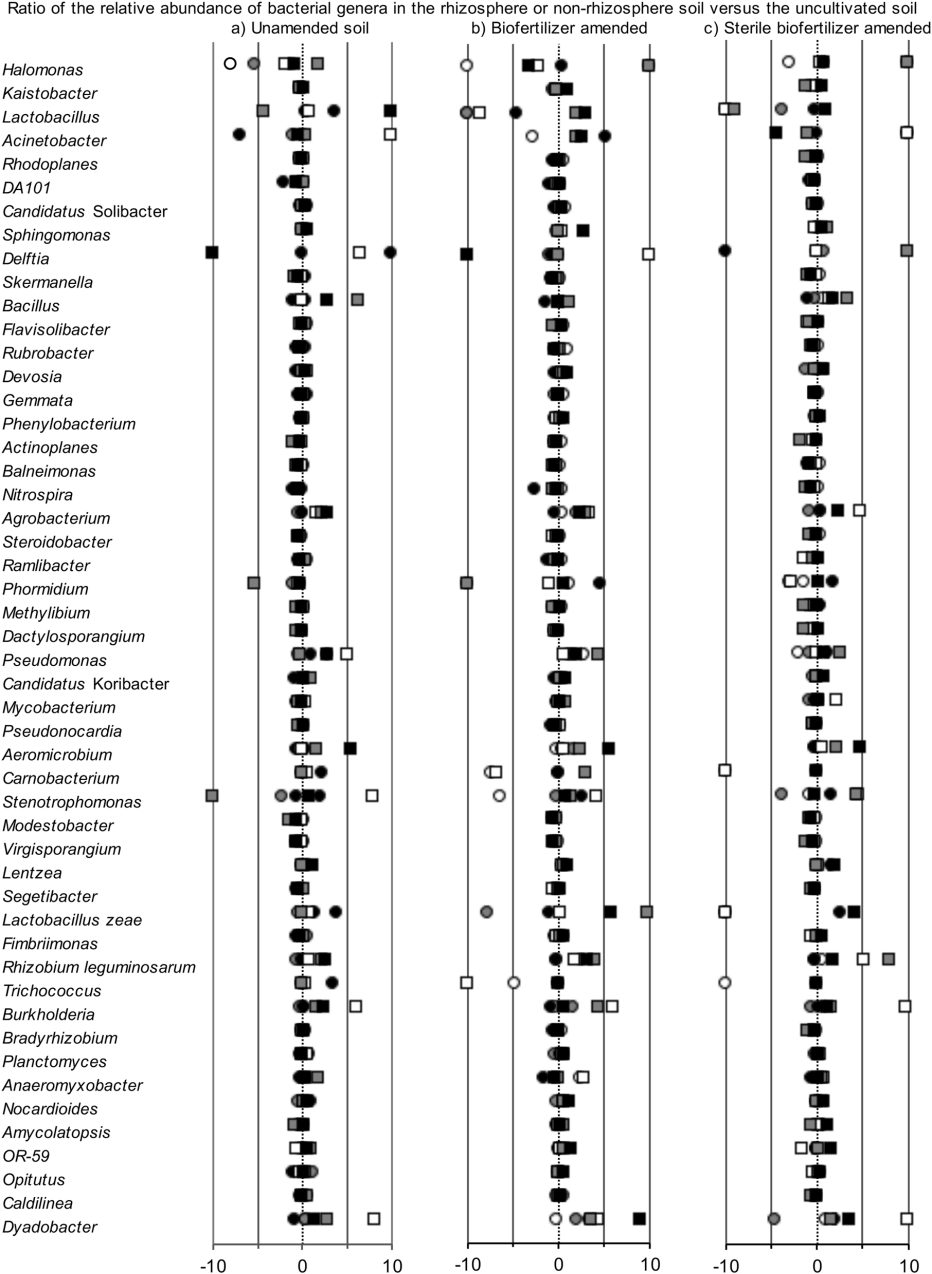
Ratio of the relative abundance of the 50 most abundant bacterial genera in the (**a**) unamended, (**b**) biofertilizer and (**c**) sterile biofertilizer amended non-rhizosphere soil after 44 days (white circle), 89 days (grey circle) and 130 days (black circle) or the rhizosphere soil after 44 days (white square), 89 days versus the uncultivated soil.

The cultivation of the maize had often a strong effect on the bacterial community in the unamended, biofertilizer or sterile biofertilizer amended soil (Fig. 4). The changes in the relative abundance of most bacterial groups in the non-rhizosphere and rhizosphere compared to the uncultivated soil were different between the unamended, biofertilizer or sterile biofertilizer amended soil, and depended on sampling time (day 44, 89 or 130) (Supplementary Table S8 online).

The PCA and PCoA (Data not shown) showed a shift in the bacterial community structure in the unamended, biofertilizer and sterile biofertilizer amended rhizosphere soil with time, but the effect of cultivation of maize was smaller than that of time (Supplementary Figs. S5, S6, S7 online). Although the perMANOVA analysis indicated often a significant effect of the cultivation of maize on the bacterial community structure in the unamended, biofertilizer and sterile fertilizer amended soil, the effect was always smaller than the effect of time, except when considering all OTUs in the unamended soil.

### The combined effect of application of biofertilizer and cultivation of maize on the bacterial community structure

**Figure 5:**
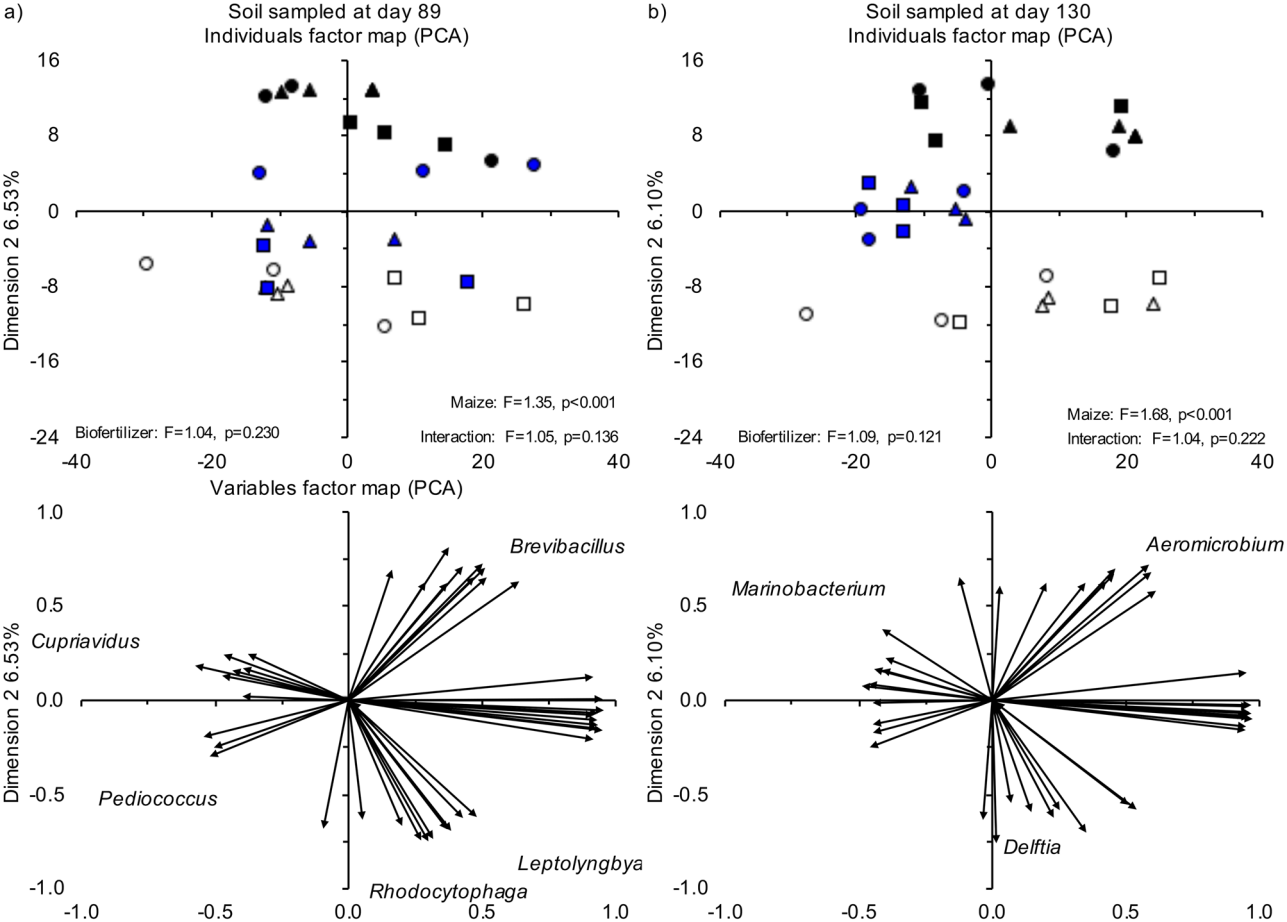
Principal component analysis (PCA) with all the bacterial groups assigned up to the taxonomic level of genus after (**a**) 89 days and (**b**) 130 days in the uncultivated unamended soil (white square), uncultivated soil amended with biofertilizer (white circle) and uncultivated soil amended with sterile biofertilizer (white triangle), unamended non-rhizosphere soil (blue square), biofertilizer amended non-rhizosphere soil (blue circle) and sterile biofertilizer amended non-rhizosphere soil (blue triangle), and unamended rhizosphere soil (black square), biofertilizer amended rhizosphere soil (black circle) and sterile biofertilizer amended rhizosphere soil (black triangle).

A PCA and PCoA separated the bacterial community in the rhizosphere from that in the uncultivated soil while the application of biofertilizer or sterile biofertilizer did not separate the bacterial communities after 89 or 130 days (Fig. 5, Supplementary Figs. S8, S9, S10 online). Cultivation of maize (uncultivated, non-rhizosphere and rhizosphere soil) had always a higher significant effect on the bacterial community structure than treatment (unamended soil, or soil amended with biofertilizer or sterile fertilizer). The effect of cultivation of maize was also more significant in the order 89 < 130 days as indicated by the F and *P* values.

## Discussion

The biofertilizer was obtained from a local farmer in one of the high maize yields producing parts of Mexico, i.e. Valley of the Mesquital in Hidalgo. The farmer applied this biofertilizer to maize and found increased yields. In a greenhouse experiment reported here, the amount and timing of the biofertilizer application in the field were copied as good as possible.

The biofertilizer had some characteristics that might limit its possible prolonged application to cultivated crops. First, the EC was high so a repetitive application during different crop cycles might increase the soil salt content and ultimately inhibit plant growth. Second, the high NH_4_^+^ content will benefit crop growth. However, high and repetitive applications of the biofertilizer might lead to excess mineral N in soil which might favour NO_3_^−^ leaching and emissions of N_2_O, i.e. a greenhouse gas.

The bacterial community structure in the biofertilizer was dominated by different Lactobacillales, e.g. *Lactobacillus, Carnobacterium* and *Trichococcus*. Lactic acid bacteria, associated with various plant and animal niches include industrially important genera, are Gram-positive microaerophilic bacteria that ferment hexose sugars to produce primarily lactic acid^15^. Lactic acid bacteria have been found to have a wide range of plant growth promoting characteristics. Some lactic acid bacteria can increase nutrient availability from compost and other organic material and are biocontrol agents of a wide variety of fungal and bacterial phytopathogens^8^. They can stimulate plant growth and have been shown to produce indole-3-acetic acid (IAA)^16^. Giassi et al.^17^ reported that they solubilize phosphate, and some strains of lactic acid bacteria from a sugarcane ferment can fix atmospheric nitrogen. Javaid^18^ applied a biofertilizer with lactic acid bacteria to a farmyard manure amended soil that improved root and shoot growth in rice (*Oriza sativa *L.). Limanska et al.^19^ reported that *Lactobacillus plantarum* increased germination, shoot and root length of tomatoes (*Lycopersicon esculentum*). Quattrini et al^20^ used *L. plantarum* that exhibited as biocontrol of fungi and induced cereal germination and promoted plant growth.

It has been reported that application of biofertilizers to crops increases plant development sometimes, but mostly no effect was found. Mayer et al.^21^ applied two types of biofertilizer for 4 years and they found no significant effect of them on crop yields, soil microbial biomass, soil microbial activity and microbial community structure in soil. Megali et al.^22^ applied a biofertilizer to maize in a field experiment but it did not increase maize biomass. Javaid^18^ applied a biofertilizer with lactic acid bacteria to a farmyard manure amended soil in a pot experiment that improved root and shoot growth in rice (*Oryza sativa *L.), but not when applied to NPK amended soil. Nuzzo et al.^10^ studied the effect of different formulations of plant growth-promoting bacteria (Lactobacilli, Rhizobia, etc.), yeasts and mycorrhizal fungi on growth of tomato plants (*Solanum lycopersicum* L.), but they did not affect plant growth. However, when a positive effect of biofertilizer on crop growth was reported, it was not due to the added microorganisms, but due to the substrate used^23^. Mayer et al.^21^ used sterilized treatments in the application of a microbial inoculant in a field experiment and did not find significant differences between sterilized and unsterilized treatments.

It is difficult to pinpoint the factor that might stimulate plant growth when a biofertilizer is applied to soil. A potential increase in plant development might be due to the activity of the microorganisms applied with the biofertilizer, i.e. plant growth promoting bacteria, the nutrient content of the biofertilizer, e.g. inorganic N, or another factor. A biofertilizer applied to soil to stimulate plant growth often contains nutrients, mostly mineral N as in this study, to guarantee the survival of the microorganisms. Application of a biofertilizer without nutrients might reduce plant growth as the microorganism in the biofertilizer might compete for nutrients with the plants. Microorganisms are more dispersed in the soil matrix than plant roots, so they more easily can immobilize mineral N inhibiting plant development. A biofertilizer, therefore, normally contains plant nutrients. As such, if a positive effect of a biofertilizer on plant development is detected it might be due to the nutrients in the biofertilizer and not due to the activity of the microorganisms. As such, it is difficult to distinguish between the effect of the microorganisms, the substrate or a combination of both. Therefore, an experiment without the inclusion of a sterilized biofertilizer makes it impossible to state if the microorganisms affected plant growth or the substrate in which the microorganisms were contained. Sterilizing the biofertilizer as done in this experiment will allow to separate a possible effect of the microorganisms on plant development and the possible effect of the substrate.

Application of biofertilizer or sterile biofertilizer had no effect on plant development. As such, the bacteria in the biofertilizer had no effect on plant development and different factors can explain this. First, the soil conditions to which the microorganisms were applied, e.g. temperature, water, salt and oxygen content, and organic material composition, might be different from the environment from which the microorganisms were isolated compromising their survival. Second, microorganisms in soil are in equilibrium with their environment, resources, and predators. Applying microorganisms disrupts this equilibrium and the allochthonous microorganisms are not protected within aggregates and so easily preyed upon. Third, movement of microorganisms is limited in soil so only a limited number of them will reach the developing roots. Coating the maize seeds with microorganisms might be a better way to bridge the physical divide between microorganisms and roots. It remained to be seen if the microorganisms are “taken along” the prolonging roots and become beneficial for the plants. Fourth, the microorganisms in the biofertilizer used in this study might have no maize growth stimulating capacity although the farmer from which the biofertilizer was obtained applied it when cultivating maize and insisted they did.

Wang et al.^24^ reported that the application of an autoclaved and sterile biofertilizer had a similar effect on bacterial populations as the application of the biofertilizer to soil. This indicated that the substrate added with biofertilizer and not the microorganisms in the substrate affected the bacterial community^21,24^. In this study, the application of biofertilizer or sterilized biofertilizer had only a small effect on the bacterial community structure.

The regular application of the biofertilizer between day 44 and day 130 had a limited effect on the bacterial community structure. Additionally, only a limited number of the bacterial groups applied with the biofertilizer affected the relative abundance of these groups in soil. As such, the bacteria in the biofertilizer did not alter the bacterial community structure. It would be interesting to investigate if the bacteria in the biofertilizer would survive other experimental conditions than the ones used in this study and if the same would happen to members of a different consortium.

How bacterial groups are affected by the rhizosphere will depend on factors, such as soil conditions, e.g. temperature and water content, soil characteristics, e.g. particle size distribution, the availability and composition of the organic material, the availability of nutrients, e.g. nitrogen and phosphorus, type of plant cultivated, microbial competition and the bacterial metabolic capacities. In this study, soil conditions, i.e. water content and temperature, and soil characteristics such as particle size distribution, were the same, so soil organic matter and nutrient content were the most important differences between the uncultivated and rhizosphere soil. The release of exudates by the plants and dying roots increased the easily decomposable organic material in the rhizosphere. The rhizosphere nutrient content is different from that in the uncultivated soil as plants require nutrient such as N, P and K, for growth, and compete with soil microorganisms for them. Other soil characteristics might also be different between the rhizosphere and the uncultivated soil, but these changes can assumed to be small, e.g. pH, water content and oxygen concentration, so that their possible effect on the bacterial community structure should be small^25,26^.

Some of the bacterial genera most enriched in the rhizosphere of maize in this study are well known to be favoured by the root environment, e.g. *Agrobacterium, Burkholderia, Pseudomonas* and *Rhizobium,* e.g. Vieira et al.^27^. Members of *Burkholderia* have been described as phosphate-solubilizing bacteria^28^ and phylotypes belonging to *Burkholderia* are often enriched in the rhizosphere^29,30^. Phylotypes belonging to *Agrobacterium* live in different environments (bare soil, rhizosphere, host plants) and are often part of the core rhizosphere microbiome, e.g. Xu et al.^31^. *Rhizobium leguminosarum* is a N_2_ fixing bacteria that lives in symbiosis with legumes^32^ so might be well adapted and enriched in the rhizosphere of other plants also. Some species of *Pseudomonas,* i.e. *P. putida*, were enriched in the rhizosphere of maize as in this study^33^. However, Yang et al.^34^ reported that their relative abundance decreased in the rhizosphere. As mentioned before, not only the cultivation of a plant will determine if a bacterial group is enriched, but also environmental factors, and soil and plant characteristics.

The relative abundance of some bacterial genera was highly variable in the rhizosphere compared to the uncultivated soil over time, e.g. *Acinetobacter*, *Delftia*, *Halomonas*, *Lactobacillus*. It can be assumed that changes over time in organic material, nutrients and competition with other bacteria determined these large fluctuations, but it is not clear why members of these bacterial groups show such large variations over time. For instance, most members of *Halomonas* are halotolerant or halophilic and oligotrophic^35^, while some phylotypes that belong to *Acinetobacter* are heterotrophic nitrifiers and aerobic denitrifiers found in oligotrophic ecosystems with a high tolerance to extreme conditions^36,37^.

## Conclusion

Application of the biofertilizer or sterilized biofertilizer had no effect on maize development and a limited effect on the bacterial community structure. The bacteria applied with the biofertilizer had only a limited effect on the relative abundance of these bacteria in the soil. As such, both the bacteria in the biofertilizer or the substrate of the biofertilizer did not stimulate maize growth and did not alter the bacterial community structure. Cultivation of maize, however, had a larger effect on the bacterial community structure than application of the biofertilizer or sterile biofertilizer.

It has to be remembered that this experiment was done in a greenhouse and the application of the biofertilizer in a field experiment might be necessary to further investigate the plant growth stimulating capacity of the consortium. Additionally, it would be worthwhile to investigate the effect of the biofertilizer on other crops cultivated in different agroecosystems.

## Methods

### Field location and soil sampling

The soil used in this experiment was collected from an agricultural field cultivated with maize at the “*Instituto Tecnológico Superior del Oriente del Estado de Hidalgo*” (ITESA) located in Apan, State of Hidalgo, Mexico (19° 73′ N, 98° 46′ W). The 0–20 cm top soil layer of three 400 m^2^ plots was sampled 20 times. The soil from each plot was pooled separately so that three soil samples (*n* = 3) were obtained. This field based replication was maintained in the greenhouse experiment so as to avoid pseudo-replication. The soil samples were passed separately through a 5 mm sieve and characterized.

The soil is classified as a Phaeozem according to “World Reference Soil (WRS) system”, with pH 6.6, electrolytic conductivity (EC) 0.22 dS m^−1^ and water holding capacity (WHC) 515 g kg^−1^. The sandy clay loam soil with clay content 240 g kg^−1^, sand content 530 g kg^−1^ and silt content 230 g kg^−1^, had an ammonium content 8.16 mg kg^−1^ dry soil, nitrate 1.91 mg kg^−1^ dry soil and nitrite 0.01 mg kg^−1^ dry soil. The maize seeds were the hybrid variety 215 W obtained from Eagle^®^ Sinaloa (Mexico).

### Characteristics of the biofertilizer

Although a biofertilizer can be described in different ways we use the definition as given by^38^. Vessey defined (2003) a biofertilizer as “*a substance which contains living micro-organisms which, when applied to seeds, plant surfaces, or soil, colonize the rhizosphere or the interior of the plant and promotes growth by increasing the supply or availability of primary nutrients to the host plant*”*.* As the consortium used in this study fits the definition of a biofertilizer as given by Vessey^38^ we will refer to the consortium as the biofertilizer or when sterilized to the sterilized biofertilizer throughout the manuscript.

The “biofertilizer” used in this study was a mixture of bacteria and leachate from compost of cow manure and was obtained from a local farmer in Hidalgo (Mexico) and characterized chemically and microbiologically. The cow manure was composted on a cement floor with a small inclination so that leachate could be collected easily. The farmer adds the leachate to the mixture of the bacteria to guarantee their survival and as an additional plant nutrient source. The farmer applies this solution regularly to fertilize his fields cultivated with maize. A same application protocol and procedure was used in this study to mimic the field experiment. Half of the biofertilizer obtained from the local farmer was sterilized by autoclaving at 121 °C for 20 min on three consecutive days so as to determine the effect of the microorganisms in the biofertilizer on the maize plants and the bacterial community structure, and the effect of the nutrients added with the biofertilizer.

### Experimental design and a greenhouse experiment

The research was done in a greenhouse at Cinvestav-Zacatenco situated to the north of Mexico City (Mexico). The experiment used a completely randomized block design with six treatments. The treatments combined as a first factor soil cultivated with maize or left uncultivated. A second factor included soil amended with the biofertilizer, sterilized biofertilizer or not fertilized. The daily temperature in the greenhouse ranged from 15 °C as minimum and reached a maximum 35 °C from April to August of 2017.

As the experimental protocol was complex, a diagram of the different treatments and sampling is given in Supplementary Fig. S11 online. A total of 162 PVC columns with diameter 17 cm and height 60 cm were used in the experiment. Each pot was filled at the bottom with 0.5 kg tezontle, a highly porous volcanic rock, and 10 kg soil was added on top. The 162 columns included 6 treatments (uncultivated unamended soil, uncultivated soil amended with biofertilizer, uncultivated soil amended with sterile biofertilizer, maize cultivated unamended soil, maize cultivated soil amended with biofertilizer, maize cultivated soil amended with sterile biofertilizer; *n* = 6), 3 sampling times (day 44, day 89 and day 130; *n* = 3), three different soil samples (*n* = 3), with three columns planted with a maize plant per soil sample (*n* = 3). Three columns of each soil sample were planted with a maize plant to account for plants that might die so that at least one mature plant was obtained per treatment, sampling time and soil sample. The soil in the 162 PVC columns was adjusted to 40% WHC with distilled water and conditioned in the greenhouse for a week. Additionally, three PVC columns were filled with soil from each soil sample (*n* = 3), adjusted to 40% WHC with distilled water and conditioned for a week. These three soil samples were used to extract DNA as described below and defined the bacterial community at the onset of the experiment, i.e. time 0.

Maize seeds variety 215 W Eagle hybrid seeds^®^ were obtained from the farmer that provided us with the biofertilizer. Three washed maize seeds were planted at 3 cm depth in 81 columns, while the remaining columns were left uncultivated. Seven days after emergence, the most vigorous plantlet was kept and the other two discarded. After 44 days, the biofertilizer or the sterilized biofertilizer was diluted with water and applied with an atomizer (10 ml m^−2^ or similar to 100 l applied ha^−1^ by the farmer) so that it was added as fine spray evenly on soil of each pot when the seeds were planted. A similar volume of water was applied in the same way to the unfertilized treatment. Five more applications of the biofertilizer, sterilized biofertilizer or water by aspersion were done during the cultivation of the maize plants. As such, the uncultivated or maize plant cultivated soil was applied with the biofertilizer, sterile biofertilizer or water on 13th April, 28th May, 5th June, 13th July, 2nd August and 12th August 2017.

### Soil and plant sampling

After 44 (27th May 2017), 89 (11th July 2017) and 130 days (21st August 2017), three columns from each treatment (*n* = 6) and soil sample (*n* = 3) were selected at random. Soil was removed from each column. The cultivated and uncultivated soil was sampled, characterized, and extracted for DNA as described below. The non-rhizosphere soil was separated from the rhizosphere soil by shaken the plants gently. The soil adhered to the roots was considered the rhizosphere soil. A 20 g sub-sample of the uncultivated, non-rhizosphere and rhizosphere soil was stored at − 20 °C pending extraction of DNA, while the pH and mineral N was determined in the remaining soil. Roots and shoots were separated, weighted and their length measured. The roots and shoots were dried in an oven at 60 °C for 24 h and weighed.

### Soil physicochemical characterization

The moisture content of the soil was determined by weight loss after samples were dried at 60 °C in an oven for 24 h. The WHC was determined by saturating 50 g dry soil with distilled water, left to drain overnight and measuring the amount of water retained. The EC was measured in a soil paste (200 g soil/110 ml distilled H_2_O) with an HI 2300 microprocessor (HANNA Instruments, Woonsocket, RI, USA), while the particle size distribution was determined with the hydrometer method as described by Gee and Bauder^39^. The pH was determined in a 10 g soil–25 ml distilled water mixture with a calibrated pH meter (Denver Instrument, Bohemia, NY, USA) fitted with a glass electrode (3007281 pH/ATC Termofisher Scientific, Waltham, MA, USA).

Mineral nitrogen (NO_3_^−^, NO_2_^−^ and NH_4_^+^) was measured in the soil and biofertilizer. A 20 g soil sub-sample was extracted with 100 ml 0.5 M K_2_SO_4_ and filtered through Whatman filter paper^®^ while mineral N was measured with a SKALAR automatic analyser system (Breda, the Netherlands)^40^. A 20 g biofertilizer sub-sample was mixed with 80 ml 0.5 M K_2_SO_4_, filtered through Whatman filter paper^®^ and mineral N measured as described previously.

### DNA extraction and PCR amplification

A 5 ml sub-sample of the sterilized and unsterilized biofertilizer was centrifuged at 3500 rpm for 15 min and the supernatant removed. A 0.5 g sub-sample of soil was washed with 10 ml 0.15 mol l^−1^ sodium pyrophosphate to eliminate the humic and fulvic acids, centrifuged at 3500 rpm for 15 min and this process was repeated until the supernatant was clear^41^. The excess pyrophosphate was eliminated with 10 ml 0.15 mol l^−1^ phosphate buffer pH 8. Three different methods were used to extract DNA from the soil and the sterilized and unsterilized biofertilizer samples. The first technique was based on the method described by Green and Sambrook^42^. In the second method, cells were lysed with two lysis solutions and a thermal shock as described by Valenzuela-Encinas et al.^43^. The third method consisted of a mechanical disruption and detergent solution for cell lysis^44^. Each method was used to extract three times 0.5 g soil or 5 ml sterilized and unsterilized biofertilizer (a total of 1.5 g soil or 15 ml sterilized and unsterilized biofertilizer). The extracts from the soil and sterile or unsterilized biofertilizer were pooled separately.

The 16S rRNA gene (V3–V4 region of bacteria) was amplified using the primers 341F (5′-CCTACGGGNGGCWGCAG-3′) and 805R (5′-ACHVGGGTATCTAATCC-3′^45^. The PCR conditions were 94 °C for 5 min, followed by 25 cycles of 60 s at 94 °C, 45 s at 53 °C, and 60 s at 72 °C, with a final extension of 10 min at 72 °C. The PCR was repeated three times for each sample. After PCR amplification, the obtained products were cleaned using the FastGen Gel/PCR extraction Kit (Nippon Genetics Duren, Germany) and quantified using a Nanodrop 3300 fluorospectrometer (TermoFisher, Wilmington, DE, USA) with PicoGreen dsDNA. The samples were mixed in equimolar amounts and sequenced using MiSeq 300-pb paired-end runs (Illumina, CA, USA) at Macrogen Inc. (Seoul, Korea).

### 16S rDNA sequences analysis

The raw sequences were analysed with “Quantitative insights into microbial ecology pipeline” (QIIME) software (version 1.9.1)^46^. The barcode reads were demultiplexing removed from the sequences using the script extract_barcodes.py. The chimeric sequences were identified using “identify_chimeric_seqs.py” with the usearch61 method and removed^47^. The taxonomic assignment was done using the Ribosomal Data Project (rdp)^48^, against the Greengenes 16S rRNA database with a 0.8 confidence^49^. The sequences were clustered as operational taxonomic units (OTU) at 97% similarity level with the UCLUST algorithm^47^. Sequences were aligned against the Greengenes reference database using PyNAST version 1.2.2^50^. The obtained 16S dataset was filtered, all OTUs assigned to Archaea were discarded and the dataset normalized. Alpha diversity indices (Chao1, Shannon and Simpson) were calculated from 478000 rarefied sequences with QIIME.

### Statistical analysis

All statistical analyses were done in R (R 4.0.2 GUI 1.72 Catalina build^51^). The characteristics of the maize plants (*n* = 3) obtained per plot (*n* = 3) were averaged and the sequences obtained from the replicate rhizosphere or non-rhizosphere soil were summed (*n* = 3) per plot before the statistical analysis. A non-parametric test was used to determine the effect of biofertilizer application and time on the plant and soil characteristics with the non-parametric t1way test of the WRS2 package (A collection of robust statistical methods)^52^. A non-parametric test was used to determine the effect of biofertilizer application or cultivation of maize on the bacterial alpha diversity with the non-parametric t1way test of the WRS2 package^52^. Heatmaps of the relative abundances of the bacterial groups were constructed with the pheatmap package^53^. Ordination [principal component analysis (PCA)], multivariate comparison (perMANOVA) and differential abundance (ALDEx2) was done with converted sequence data using the centred log-ratio transform test returned by the aldex.clr argument (ALDEx2 package^54^). The PCA was done with the vegan package^55^. Effect of biofertilizer application and cultivation of maize on the bacterial groups was determined using a compositional approach, i.e. analysis of differential abundance taking sample variation into account (aldex.kw argument, ALDEx2 package). A permutational multivariate analysis of variance (perMANOVA) analysis was also done with sequence counts converted using the centred log-ratio transform, i.e. aldex.clr argument (ALDEx2 package (aldex.clr(counts, mc.samples = 128, denom = "all", verbose = FALSE, useMC = FALSE)). The adonis2 argument (Vegan package) was used for the perMANOVA analysis to test the effect of cultivation of maize, time and its interaction, biofertilizer application, time and their interaction, and cultivation of maize, biofertilizer application and their interaction on the bacterial community structure (#adonis2(clrcounts ~ maize*biofertilizer, data = code, permutations = 999, method = "euclidean"). Raw counts were used as input and Monte Carlo Dirichlet instances of the *clr* transformation values were generated with the function ‘aldex.clr’ of *ALDEx2* (v.1.23.2) R package^54^. Distance pairwise matrices were calculated using the Aitchison distance and the principal coordinate analysis (PCoA) was calculated on the distance matrices with *vegan* R package^55^.

### Informed consent

Permission was obtained from the farmer to use the maize seeds he provided.

### Ethical approval

The experiment in the greenhouse complied with and was conducted as stipulated by national regulations.

## Supplementary Information

Supplementary Information 1.

Supplementary Information 2.

## Data Availability

The sequences generated in this study were submitted to the NCBI SRA database under bioproject PRJNA565000.
