# Supplementary material for: Impact of a bacterial consortium on the soil bacterial community structure and maize (Zea mays L.) cultivation
Source: Sci Rep. 2021 Jun 22;11:13092. doi: 10.1038/s41598-021-92517-0 (PMC8219701; doi:10.1038/s41598-021-92517-0)
Supplement: Supplementary file 1 — Supplementary Information 1. [file 41598_2021_92517_MOESM1_ESM.pdf]

Supplementary Figures

Impact of a bacterial consortium on the soil bacterial community structure and maize (*Zea mays* L.) cultivation

Afanador-Barajas, L.N.[1,2], Navarro-Noya, Y.E.[3], Luna-Guido M.L.[1], Dendooven, L.[1*]

[1] Soil Ecology Laboratory, Cinvestav, Mexico City, Mexico,

[2] BioMat, Departamento de Ciencias Naturales, Universidad Central, Bogotá, Colombia,

[3] Cátedras CONACYT, Universidad Autónoma de Tlaxcala, Tlaxcala, Mexico

***Correspondence:** Luc Dendooven, dendooven@me.com

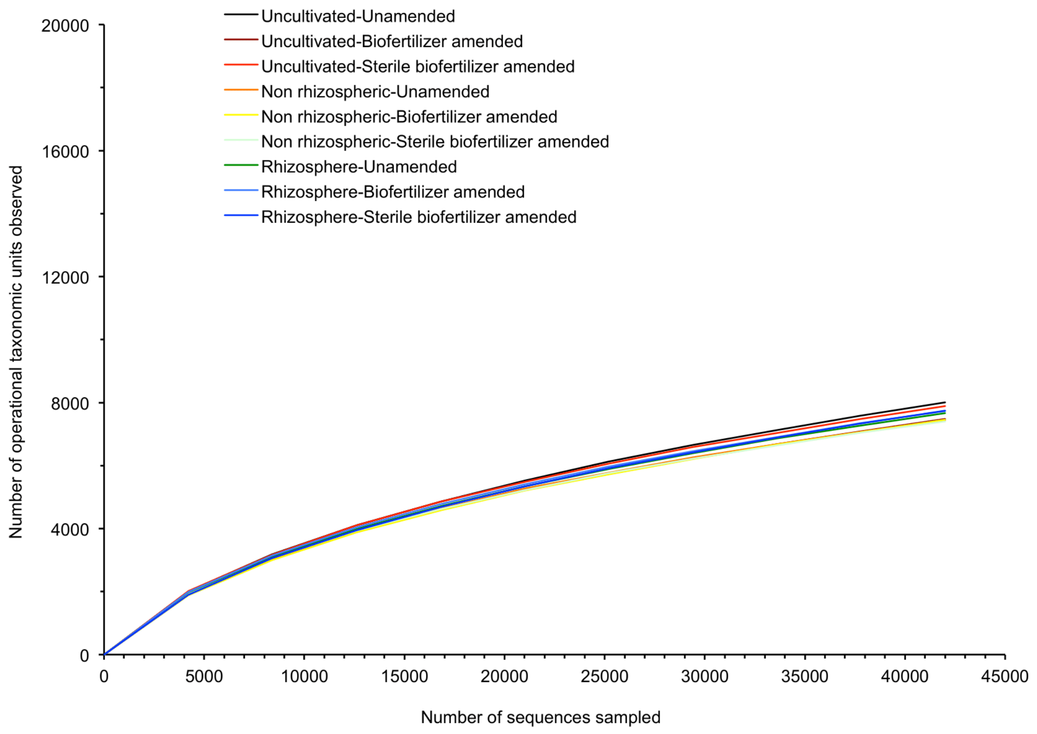

Supplementary Figure S1. Rarefication curve of the number of sequences versus the number of operational taxonomic units (OTUs)

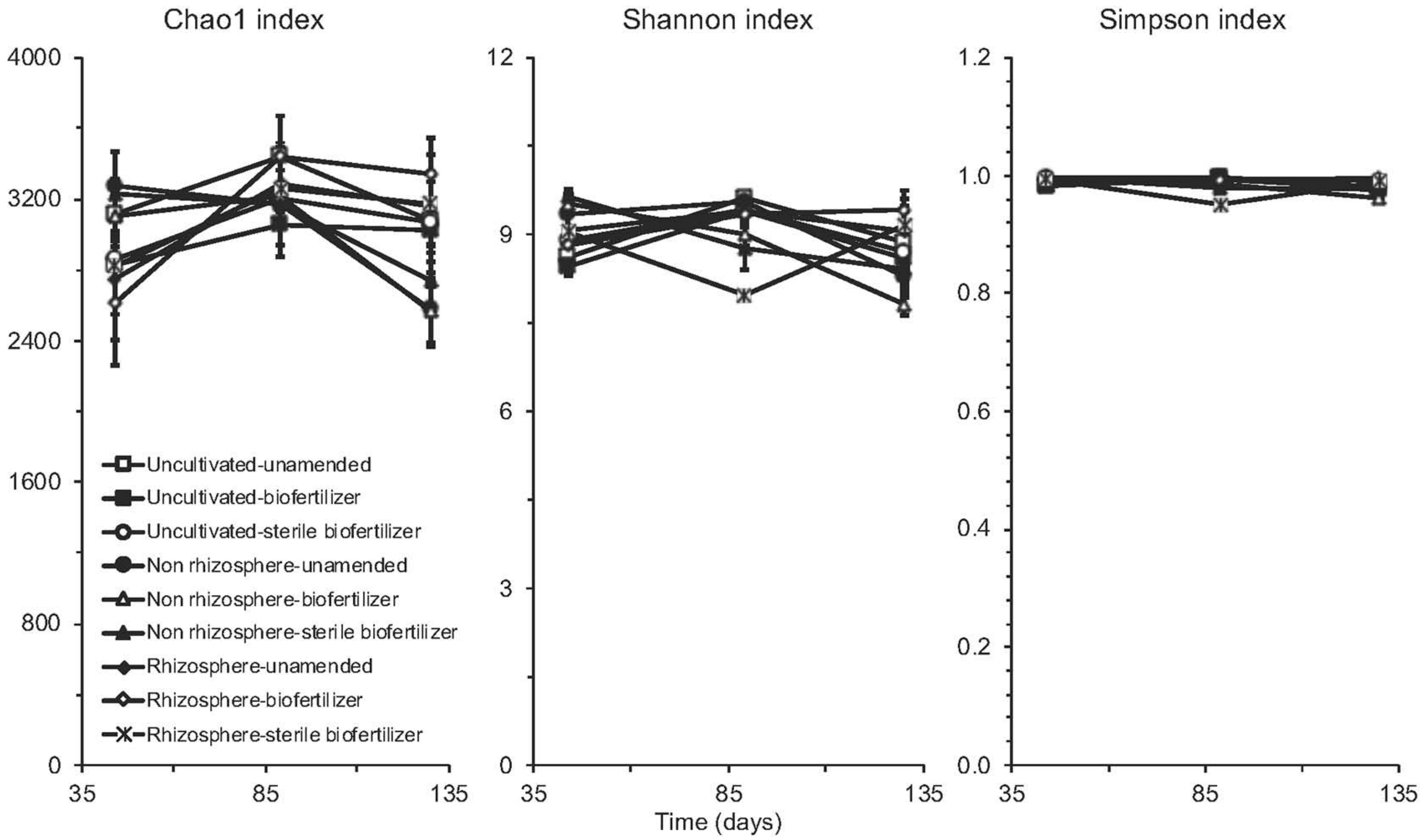

Supplementary Figure S2. Alpha diversity values in uncultivated, non-rhizosphere and rhizosphere soil of maize plants (*Zea mays* L.) left unamended or amended with biofertilizer or sterile fertilizer after 44, 89 and 130 days. Values are the mean of three measurements with bars the standard deviation of the mean.

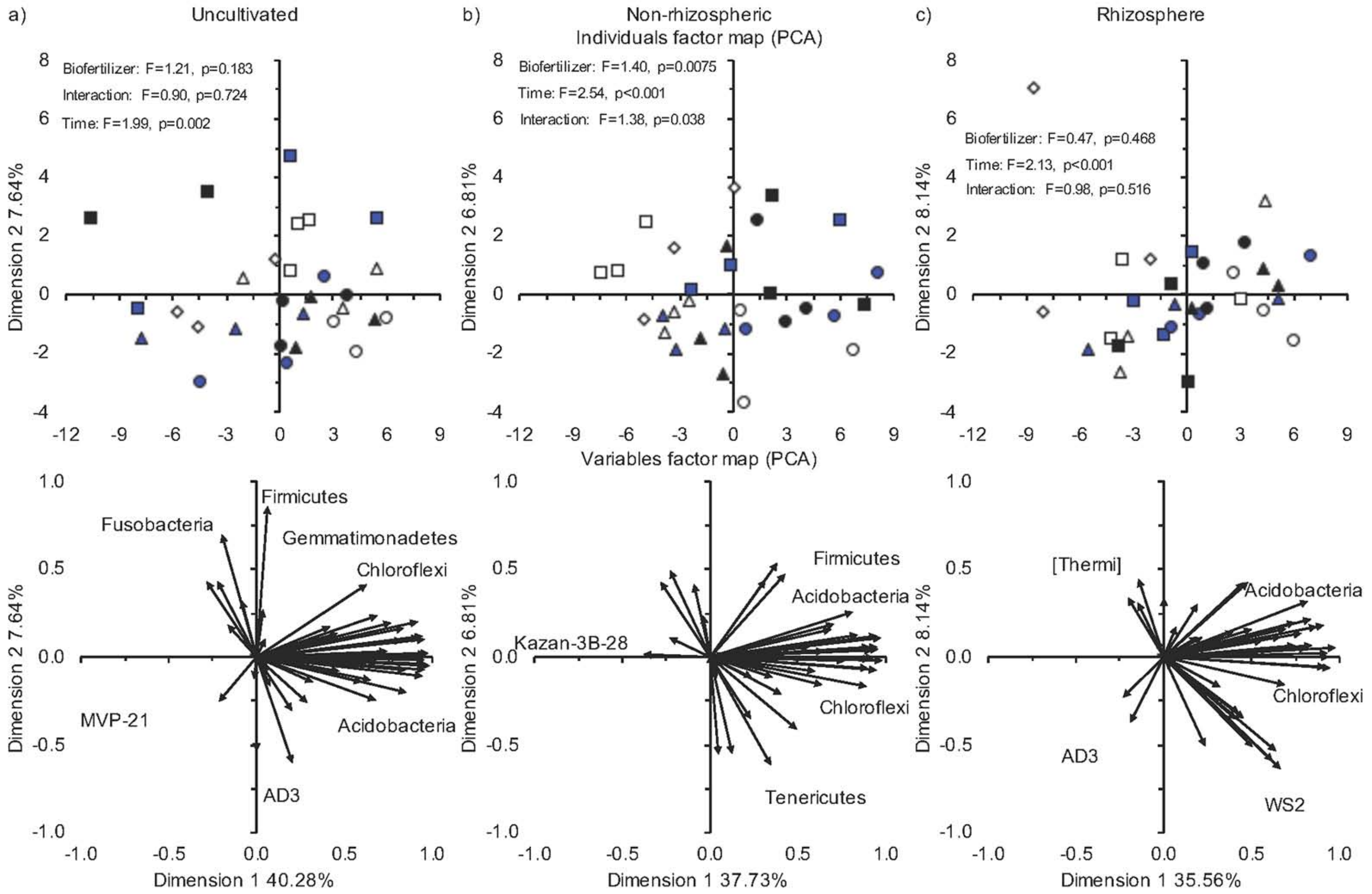

Supplementary Figure S3. Principal component analysis (PCA) with the bacterial phyla in the a) uncultivated soil, b) non-rhizosphere soil and c) rhizosphere soil at the onset of the experiment (day 0) (◇), the unamended soil at day 44 (□), day 89 (○) and day 130 (△), the soil amended with sterilized biofertilizer at day 44 (■), day 89 (●) and day 130 (▲), and soil amended with biofertilizer at day 44 (■), day 89 (●) and day 130 (▲).

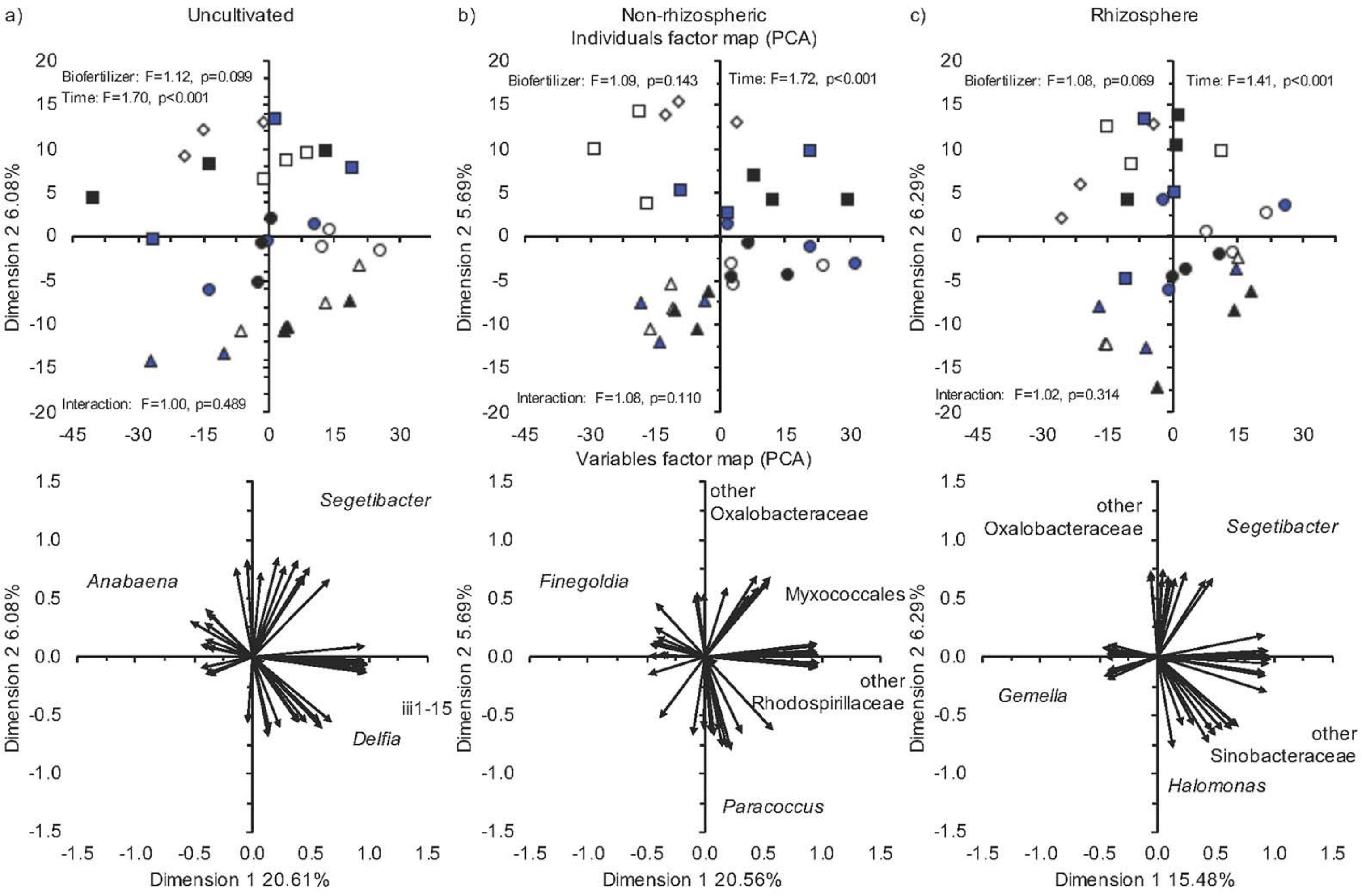

Supplementary Figure S4. Principal component analysis (PCA) with all the bacterial groups assigned to the taxonomic level of genus in the a) uncultivated soil, b) non-rhizosphere soil and c) rhizosphere soil at the onset of the experiment (day 0) (◇), the unamended soil at day 44 (□), day 89 (○) and day 130 (△), the soil amended with sterilized biofertilizer at day 44 (■), day 89 (●) and day 130 (▲), and soil amended with biofertilizer at day 44 (■), day 89 (●) and day 130 (▲)

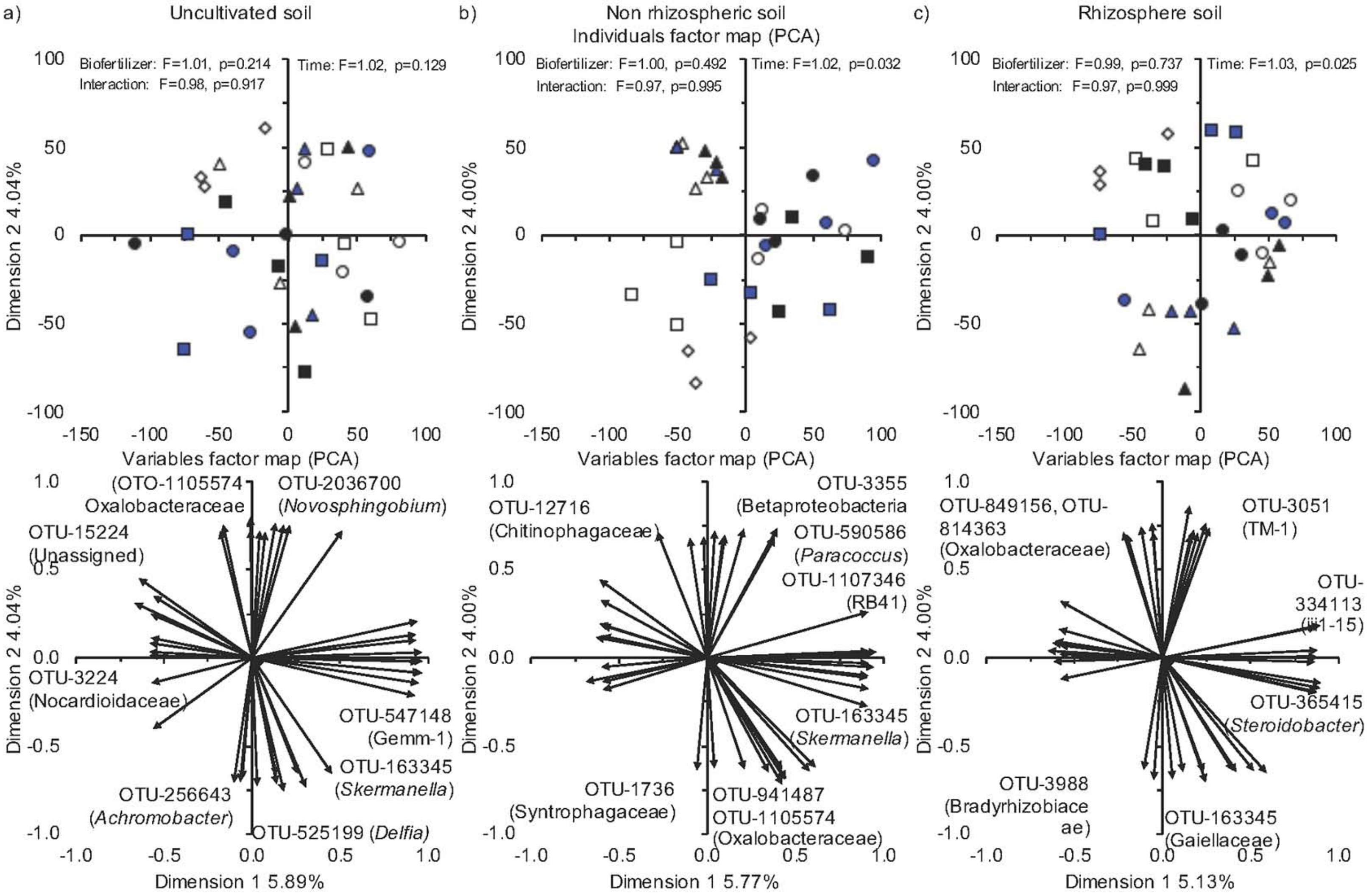

Supplementary Figure S5. Principal component analysis (PCA) with all the bacterial groups operational taxonomic units (OTUs) in the a) uncultivated soil, b) non-rhizosphere soil and c) rhizosphere soil at the onset of the experiment (day 0) (◇), the unamended soil at day 44 (□), day 89 (○) and day 130 (△), the soil amended with sterilized biofertilizer at day 44 (■), day 89 (●) and day 130 (▲), and soil amended with biofertilizer at day 44 (■), day 89 (●) and day 130 (▲).

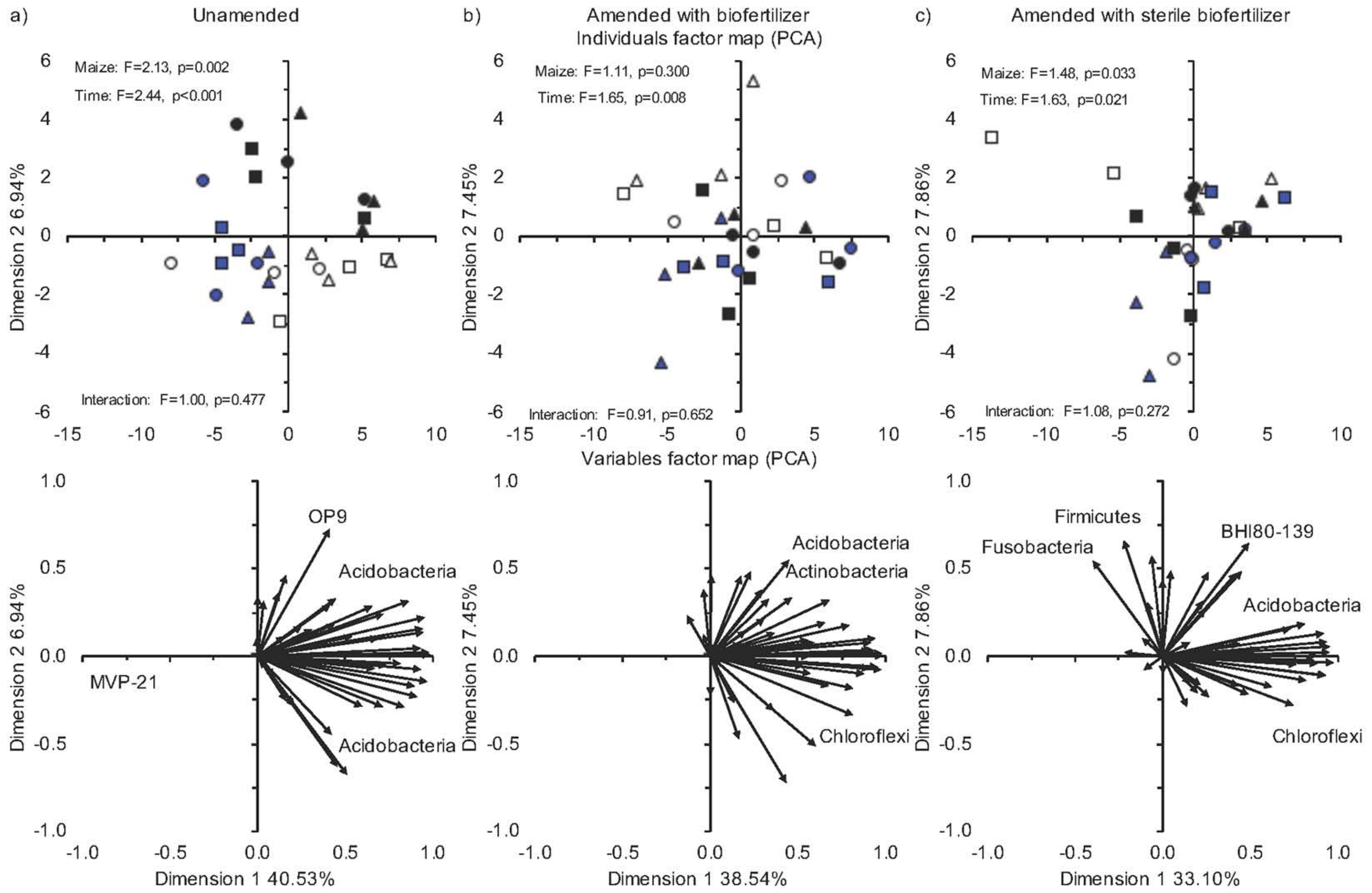

Supplementary Figure S6. Principal component analysis (PCA) with the bacterial phyla in the a) unamended soil, b) soil amended with biofertilizer soil and c) amended with sterile biofertilizer with the uncultivated soil at day 44 (□), day 89 (○) and day 130 (△), the non-rhizosphere soil at day 44 (■), day 89 (●) and day 130 (▲), and rhizosphere soil at day 44 (■), day 89 (●) and day 130 (▲).

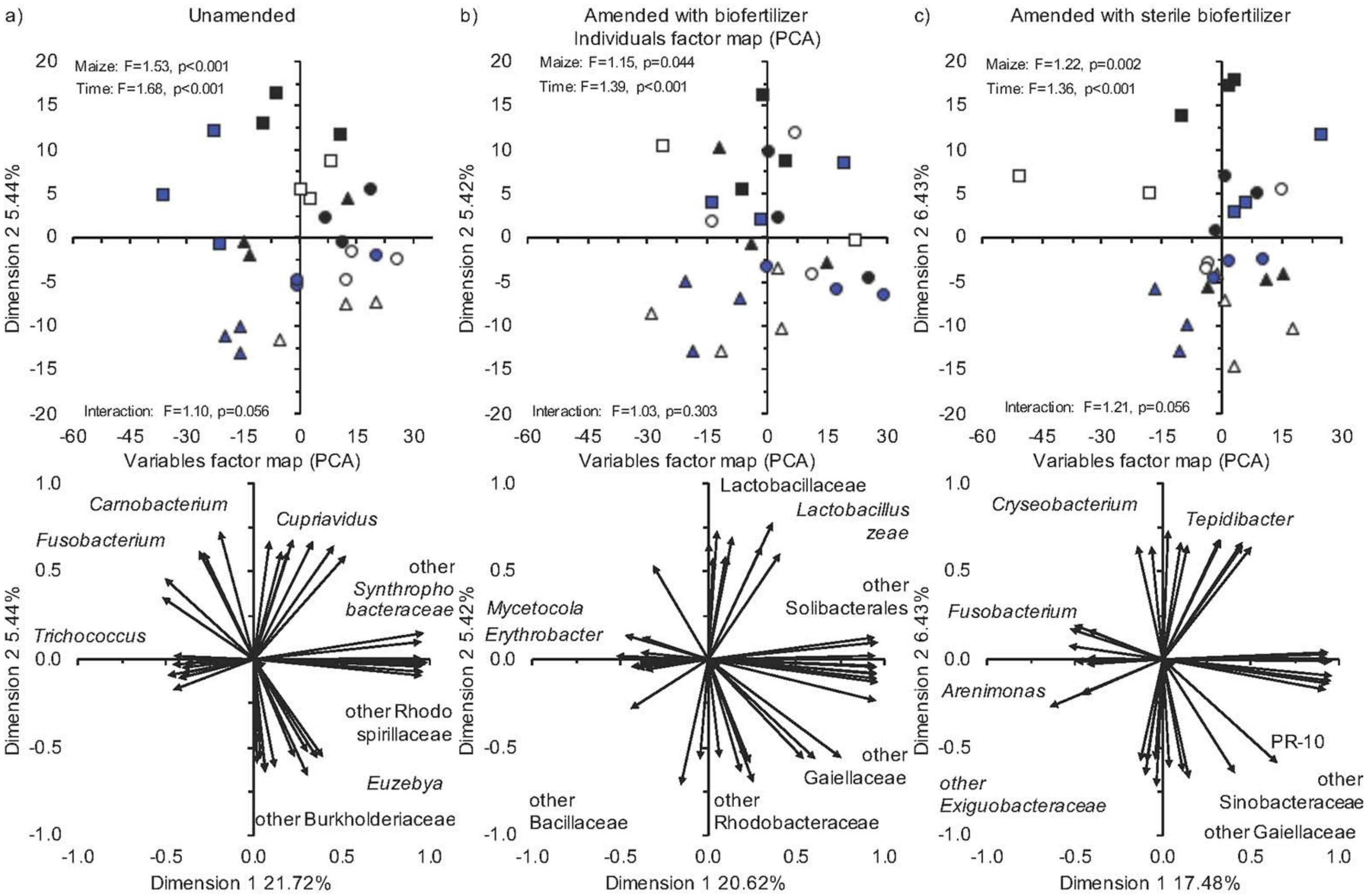

Supplementary Figure S7. Principal component analysis (PCA) with all the bacterial groups assigned to the taxonomic level of genus in the a) unamended soil, b) soil amended with biofertilizer and c) soil amended with sterile biofertilizer in soil left uncultivated at day 44 (□), day 89 (○) and day 130 (△), the non-rhizosphere soil at day 44 (■), day 89 (●) and day 130 (▲), and rhizosphere soil at day 44 (■), day 89 (●) and day 130 (▲).

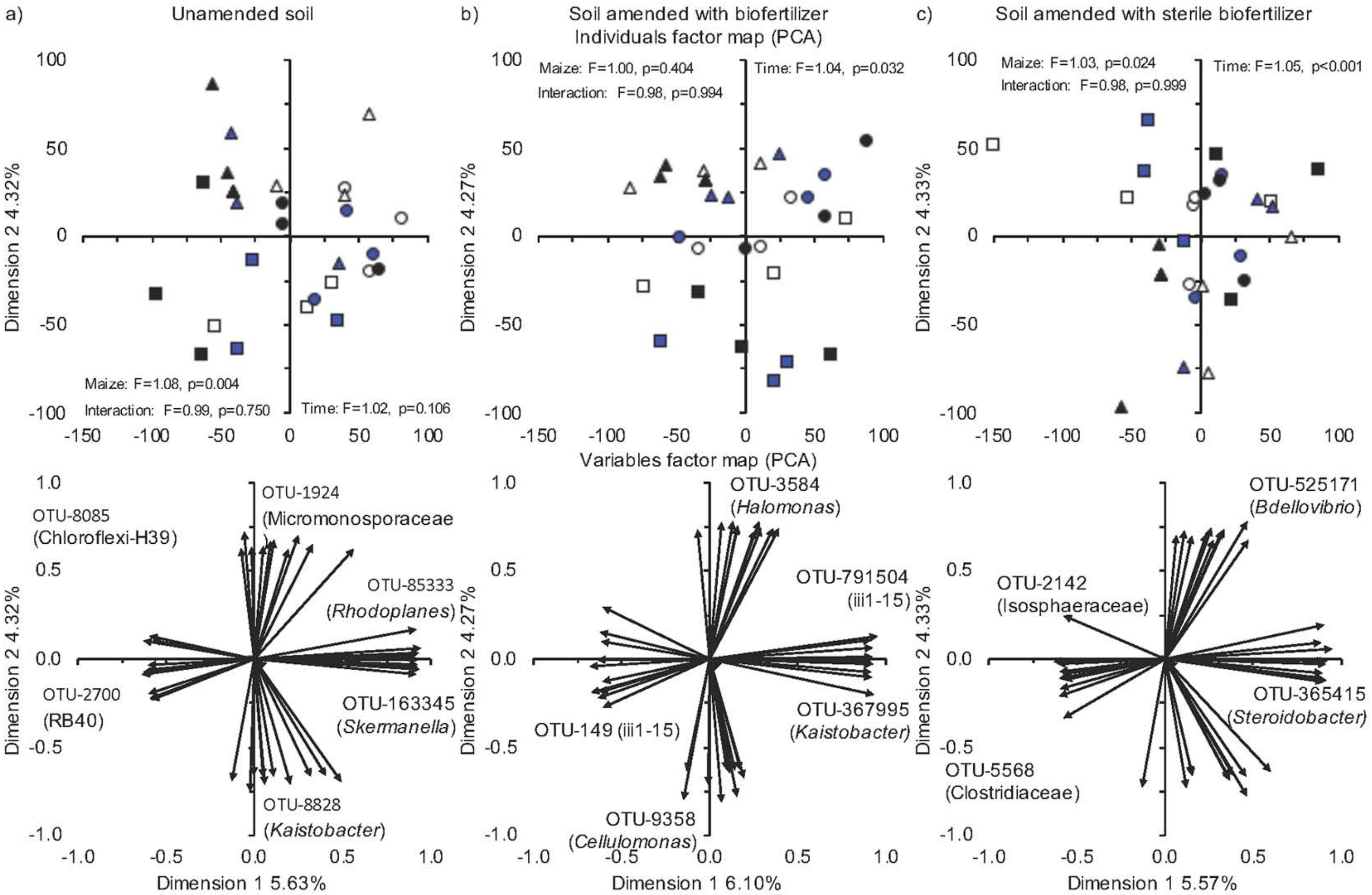

Supplementary Figure S8. Principal component analysis (PCA) with all bacterial operational taxonomic units (OTUs) in the a) unamended soil, b) soil amended with biofertilizer soil and c) amended with sterile biofertilizer in soil left uncultivated at day 44 (□), day 89 (○) and day 130 (△), the non-rhizosphere soil at day 44 (■), day 89 (●) and day 130 (▲), and rhizosphere soil at day 44 (■), day 89 (●) and day 130 (▲).

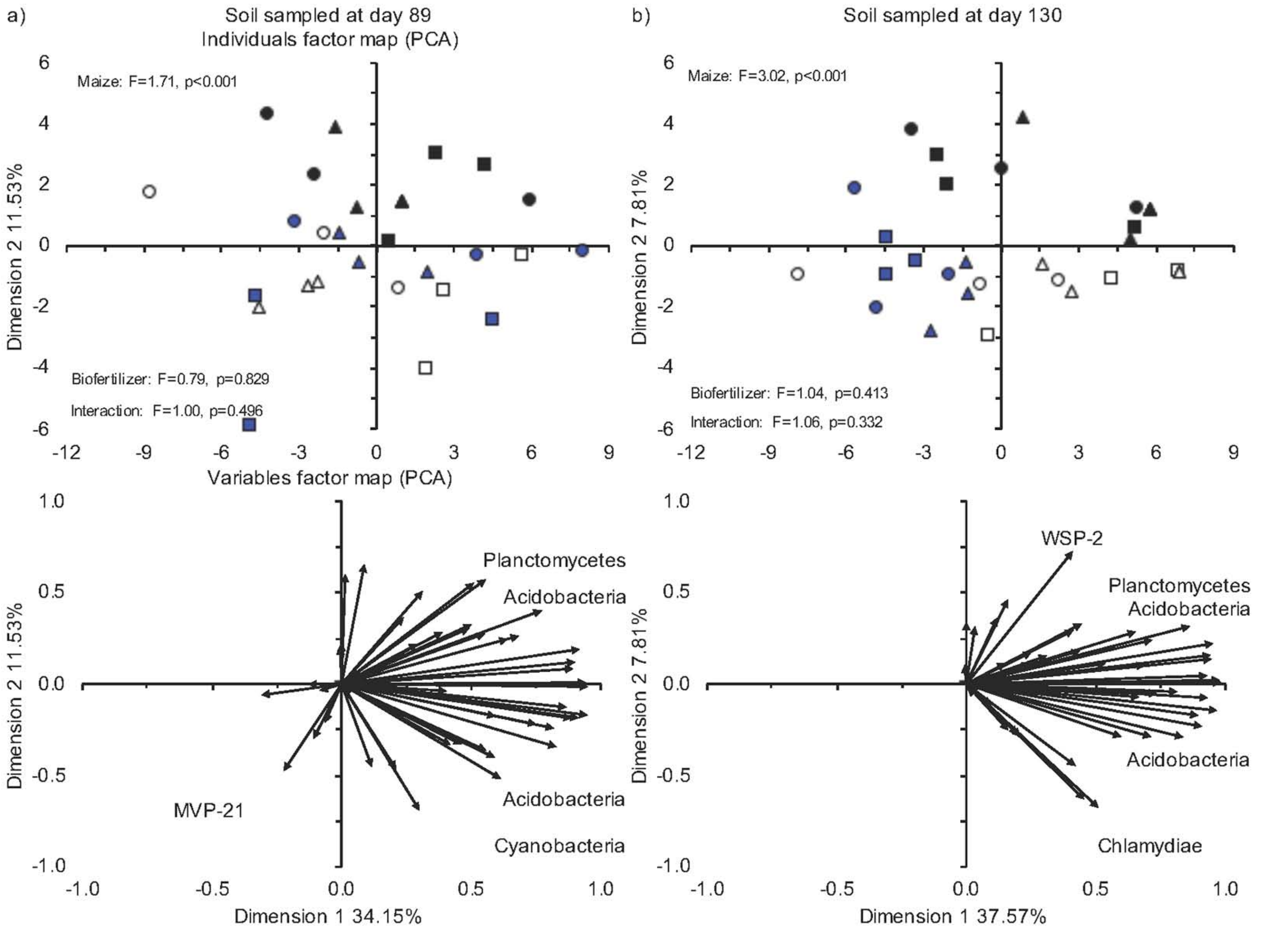

Supplementary Figure S9. Principal component analysis (PCA) with the bacterial phyla after a) 89 days and b) 130 days in the uncultivated unamended soil (□), uncultivated soil amended with biofertilizer (○) and uncultivated soil amended with sterile biofertilizer (△), unamended non-rhizosphere soil (■), biofertilizer amended non-rhizosphere soil (●) and sterile biofertilizer amended non-rhizosphere soil (▲), and unamended rhizosphere soil (■), biofertilizer amended rhizosphere soil (●) and sterile biofertilizer amended rhizosphere soil (▲).

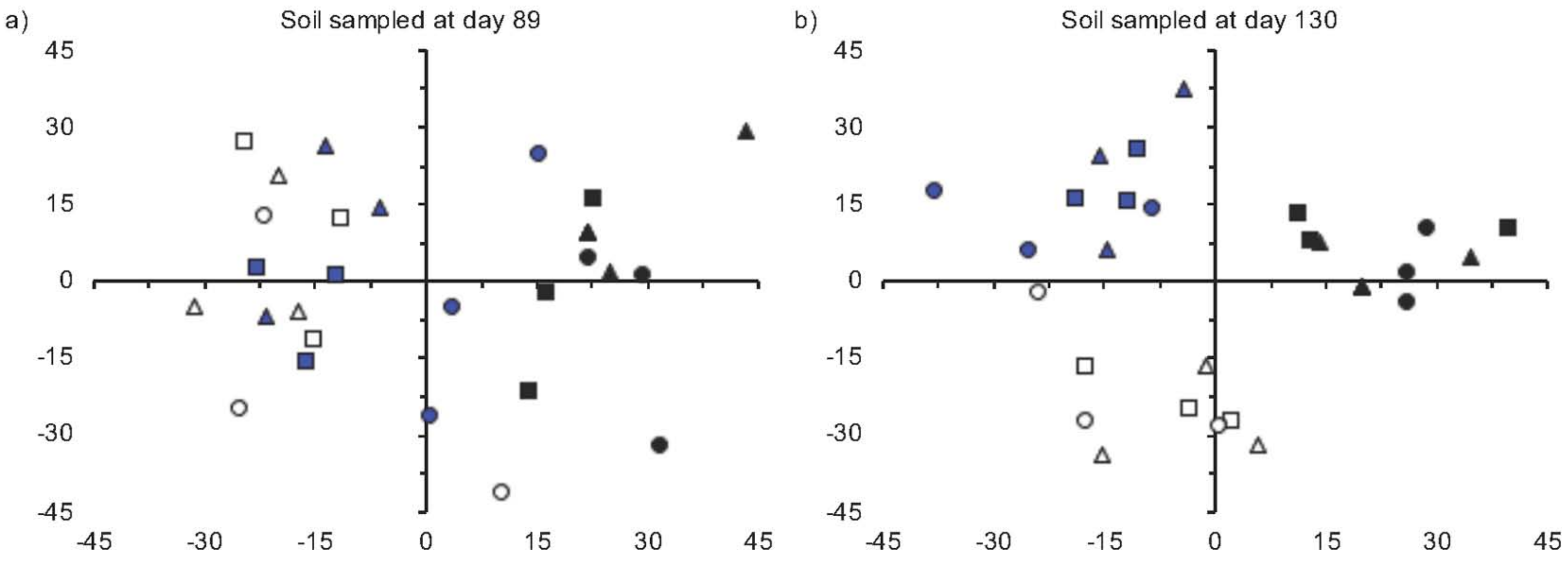

Supplementary Figure S10. The principal coordinate analysis (PCoA) using the Aitchison distance with all the bacterial groups assigned up to the taxonomic level of genus after a) 89 days and b) 130 days in the uncultivated unamended soil (□), uncultivated soil amended with biofertilizer (○) and uncultivated soil amended with sterile biofertilizer (△), unamended non-rhizosphere soil (■), biofertilizer amended non-rhizosphere soil (●) and sterile biofertilizer amended non-rhizosphere soil (▲), and unamended rhizosphere soil (■), biofertilizer amended rhizosphere soil (●) and sterile biofertilizer amended rhizosphere soil (▲).

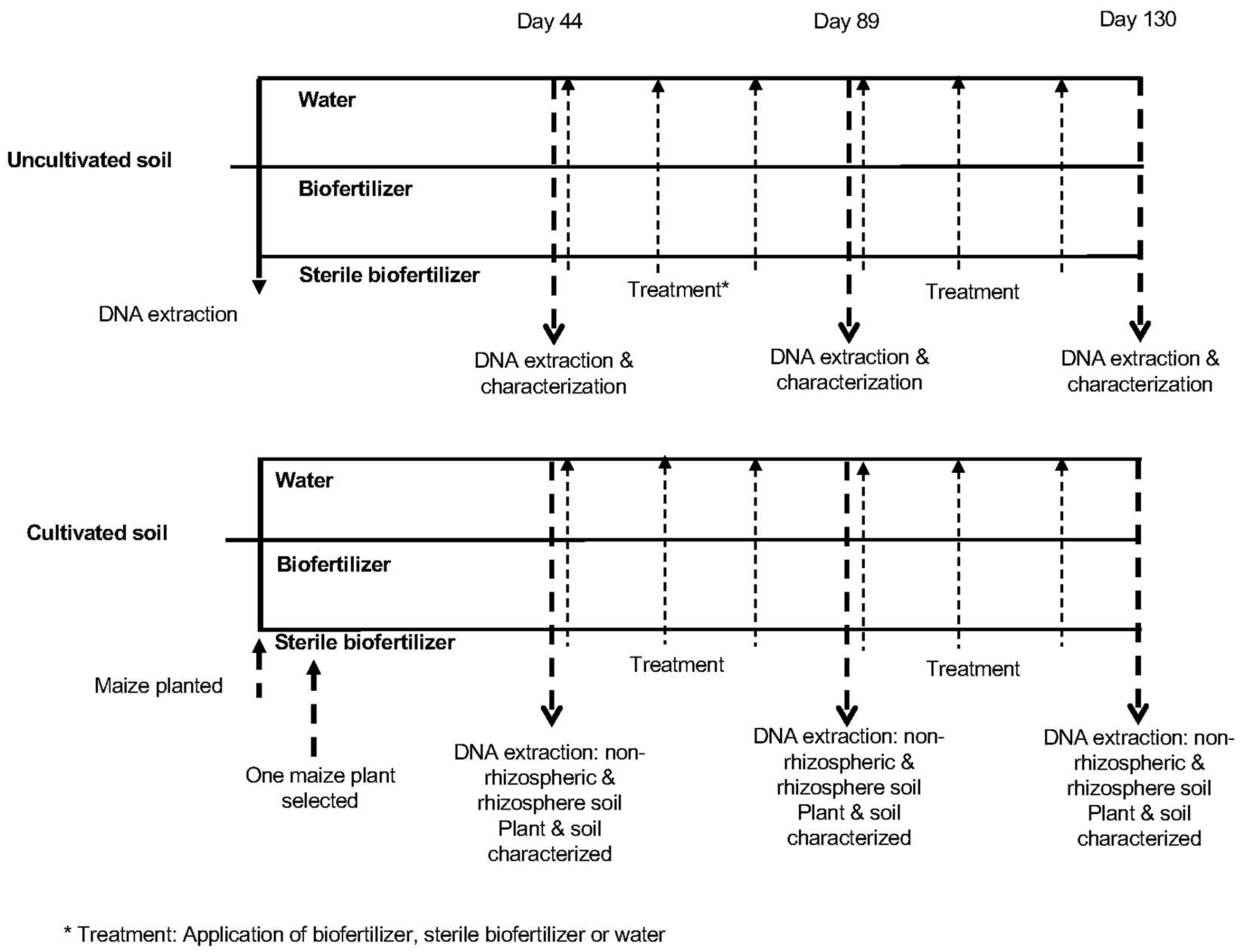

Supplementary Figure S11. Experimental design and sampling strategy.
