# Supplementary material for: Impact of a bacterial consortium on the soil bacterial community structure and maize (Zea mays L.) cultivation
Source: Sci Rep. 2021 Jun 22;11:13092. doi: 10.1038/s41598-021-92517-0 (PMC8219701; doi:10.1038/s41598-021-92517-0)
Supplement: Supplementary file 2 — Supplementary Information 2. [file 41598_2021_92517_MOESM2_ESM.pdf]

Supplementary Tables

Impact of a bacterial consortium on the soil bacterial community structure and maize (*Zea mays* L.) cultivation

Afanador-Barajas, L.N.[1,2], Navarro-Noya, Y.E.[3], Luna-Guido M.L.[1], Dendooven, L.[1*]

[1] Soil Ecology Laboratory, Cinvestav, Mexico City, Mexico,

[2] BioMat, Departamento de Ciencias Naturales, Universidad Central, Bogotá, Colombia,

[3] Cátedras CONACYT, Universidad Autónoma de Tlaxcala, Tlaxcala, Mexico

***Correspondence:** Luc Dendooven, dendooven@me.com

Supplementary Table S1. Some characteristics of the biofertilizer used.

	EC ($dS\ m^{-1}$)	pH	Ammonium	Nitrate ($mg\ kg^{-1}$)	Nitrite
Biofertilizer	20.4	6.8	864	13.5	9.0

Supplementary Table S2. Some selected characteristics of the uncultivated soil left unamended or amended with the sterilized or unsterilized biofertilizer after 44, 89 or 130 days.

	pH					Concentration of ammonium (mg NH_4^+-N kg^{-1})				
	Sampling days					Sampling days				
	44	89	130	F value	*P* value	44	89	130	F value	*P* value
Unamended	7.0 [a] A [b] a [c]	7.2 A a	6.9 A a	0.39	0.705	4.1 B a	5.3 A a	5.5 A a	8.80	0.050
Amended	6.9 A a	7.0 A a	6.9 A a	0.09	0.919	4.3 B a	1.7 A c	3.4 B b	44.80	0.002
Sterilized	6.8 A ab	6.5 A b	7.2 A a	12.59	0.024	5.4 A a	1.8 A a	3.8 AB a	17.60	0.050
F value	0.30	5.88	2.08			14.75	5.40	14.90		
P value	0.758	0.086	0.258			0.015	0.102	0.015		
	Concentration of nitrite (mg NO_2^--N kg^{-1})					Concentration of nitrate (mg NO_3^--N kg^{-1})				
Unamended	0.11 A a	0.68 A a	0.13 A a	6.46	0.089	10.2 A b	27.3 A ab	35.8 A a	18.93	0.016
Amended	0.11 A a	0.25 A a	0.19 A a	2.79	0.204	8.4 A b	20.5 A a	20.4 A ab	20.78	0.017
Sterilized	0.10 A a	0.23 A a	0.15 A a	3.85	0.154	6.7 A b	34.6 A a	22.6 A a	36.43	0.010
F value	0.07	4.08	5.64			3.09	4.67	4.90		
P value	0.935	0.119	0.082			0.163	0.105	0.087		

[a] Mean of three samples, [b] Values with the same capital letter are not significantly different between the treatments, i.e. within the columns, [c] values with the same letter are not significantly different over time, i.e. within the rows. A non-parametric test was used, i.e. t1way test of the WRS2 package, to test the effect of time and treatment on soil characteristics (A collection of robust statistical methods, Mair and Wilcox[1]) ($P < 0.05$).

Supplementary Table S3. Some selected characteristics of the soil cultivated with maize (*Zea mays* L.) left unamended or amended with the sterilized or unsterilized biofertilizer after 44, 89 or 130 days.

	pH					Concentration of ammonium (mg NH_4^+-N kg^{-1})				
	Sampling days					Sampling days				
	44	89	130	F value	*P* value	44	89	130	F value	*P* value
Unamended	7.1[a] A[b] c[c]	7.6 A b	8.4 A a	86.76	0.001	5.6 A a	2.6 A c	3.9 A b	65.87	0.002
Amended	7.0 A a	7.4 A a	7.9 A a	4.03	0.142	7.8 A a	0.7 A b	4.2 A ab	23.85	0.016
Sterilized	6.7 A b	8.1 A a	8.2 A a	11.78	0.024	5.3 A	1.6 A	4.6 A	13.61	0.050
F value	1.24	3.83	1.25			2.38	5.18	1.50		
P value	0.389	0.121	0.412			0.226	0.086	0.337		
	Concentration of nitrite (mg NO_2^--N kg^{-1})					Concentration of nitrate (mg NO_3^--N kg^{-1})				
Unamended	0.02 A a	0.25 A a	0.15 A a	< 0.01	1.000	1.3 A a	2.7 A a	2.4 A a	1.11	0.441
Amended	0.08 A a	0.31 A a	0.18 A a	10.75	0.050	0.8 A a	3.3 A a	3.3 A a	8.10	0.050
Sterilized	0.12 A a	0.20 A a	0.07 A a	2.89	0.168	0.7 A b	3.1 A ab	3.5 A a	8.23	0.039
F value	< 0.01	1.70	3.05			0.27	0.13	3.61		
P value	1.000	0.306	0.170			0.775	0.884	0.153		

[a] Mean of three samples, [b] Values with the same capital letter are not significantly different between the treatments, i.e. within the columns, [c] values with the same letter are not significantly different over time, i.e. within the rows. A non-parametric test was used, i.e. t1way test of the WRS2 package, to test the effect of time and treatment on soil characteristics (A collection of robust statistical methods, Mair and Wilcox[1]) ($P < 0.05$).

Supplementary Table S4. Effect of treatment (unamended, or amended with biofertilizer or sterile fertilizer) and plant cultivation (uncultivated, bulk and rhizosphere soil of maize plants (*Zea mays* L.)) on pH and concentrations of mineral N.

	pH		Ammonium (NH_4^+)		Nitrite (NO_2^-)		Nitrate (NO_3^-)	
Factor	F value [a]	*P* value	F value	P value	F value	P value	F value	*P* value
Plant cultivation	20.27	< 0.001	0.08	0.775	1.67	0.219	47.55	< 0.001
Treatment	2.10	0.390	1.53	0.495	2.14	0.380	1.74	0.462
Interaction plant cultivation*Treatment	2.19	0.375	1.55	0.492	0.78	0.695	2.17	0.387

[a] A non-parametric test was used, i.e. t2way test of the WRS2 package (A collection of robust statistical methods, Mair and Wilcox[1]), to test the effect of plant cultivation and treatment.

Supplementary Table S5. Effect of treatment (unamended soil, or soil amended with biofertilizer or sterile fertilizer) and plant cultivation (uncultivated, bulk and rhizosphere soil of maize plants (*Zea mays* L.)) on alpha diversity indexes.

		Chao1		Shannon		Simpson	
Factor	Days	F value [a]	*P* value	F value	*P* value	F value	*P* value
Treatment	89	0.68	0.525	7.61	0.050	7.56	0.050
	130	0.06	0.945	0.07	0.930	0.19	0.826
Plant cultivation	89	2.47	0.142	4.04	0.054	2.91	0.108
	130	22.59	< 0.001	17.01	< 0.001	15.89	< 0.001

[a] A non-parametric test was used, i.e. t1way test of the WRS2 package (A collection of robust statistical methods, Mair and Wilcox[1]), to test the effect of plant cultivation and treatment on the alpha biodiversity parameters.

Supplementary Table S6. Effect of biofertilizer application (unamended, or biofertilizer or sterile biofertilizer amended) on bacterial groups in the uncultivated, non-rhizosphere and rhizosphere soil and the effect of cultivation of maize in the unamended, biofertilizer and sterile biofertilizer amended soil using a compositional approach, i.e. analysis of differential abundance taking sample variation into account (ALDEx2 package[2]).

	Day 44	Day 89	Day 130
		Effect of biofertilizer application on bacterial groups	
Uncultivated		*Stenotrophomonas* (0.042) [a]	*Delftia* (0.029), *Ochrobactrum* (0.043)
Non rhizosphere		*Acinetobacter* (0.028)	
Rhizosphere		*Sphingomonas* (0.044)	
		Effect of maize cultivation on bacterial groups	
Unamended	*Agrobacterium* (0.049), *Devosia* (0.030), *Kaistobacter* (0.049)	*Halomonas* (0.027), *Paenibacillus* (0.047) *Stenotrophomonas* (0.042)	Cyanobacteria (0.048), Firmicutes (0.047), Tenericutes (0.046), *Aeromicrobium* (0.042), *Asteroleplasma* (0.039), *Delftia* (0.027), *Halomonas* (0.029), *Lactobacillus zeae* (0.047)
Biofertilizer	*Acinetobacter* (0.028), *Halomonas* (0.027), *Paracoccus marcusii* (0.049), *Phormidium* (0.041), *Rhizobium leguminosarum* (0.049), *Stenotrophomonas* (0.042)	*Acinetobacter* (0.030), *Lactobacillus zeae* (0.042)	*Aeromicrobium* (0.042), *Halomonas* (0.034), *Luteolibacter* (0.037), *Skermanella* (0.049)
Sterile biofertilizer	*Acinetobacter* (0.028), *Phormidium* (0.041), *Rhizobium leguminosarum* (0.049), *Tepidibacter* (0.037)	*Citromicrobium* (0.034), *Dyadobacter* (0.028), *Kaistobacter* (0.042), *Phormidium* (0.029), *Sphingomonas* (0.027)	Tenericutes (0.044), *Delftia* (0.027), other *Pseudomonas* (0.039), *Pseudomonas pseudoalcaligenes* (0.047), *Vibrio metschnikovii* (0.029)

[a] Value between parenthesis: the expected values of the Kruskal-Wallis test for each feature obtained with aldex.kw argument with converted sequence data using the centred log-ratio transform test returned by the aldex.clr argument (ALDEx2 package).

Supplementary Table S7. Effect of the application of the application of biofertilizer on bacterial OTUs in the uncultivated, non-rhizospheric and rhizosphere soil. Only the bacterial OTUs with a ratio of the relative abundance of the OTU in the biofertilizer amended soil versus the relative abundance of the OTU the unamended soil > 2 or when the OTU was not detected in the unamended soil are given.

Uncultivated	OTU-48811 *Cellvibrio* (2.1) [a]; **OTU-347529 *Turicibacter*** [b] (2.1); OTU-545507 Comamonadaceae (2.1); OTU-221349 N1423WL (2.3); OTU-3851582 (3.1), New 0 CleanUp Reference OTU-268 (3.4), OTU-604966 (5.9), **OTU-716286** (6.3), **OTU-794205** *Lactobacillus* (10.5); OTU-716006 *Lactococcus* (3.4), OTU-3013 *Halomonas* (4.2), OTU-5426 *Lactobacillus zeae* (4.2); New 0 CleanUp Reference OTU-5497 Cystobacterineae (7.3)
Non rhizosphere	OTU-813418 Sphingomonadaceae (2.3); OTU-841170 Mycoplana (3.3); OTU-579049 Bacillales (3.7); New 0 CleanUp Reference OTU-1688 Gaiellaceae (4.0); OTU-542842 (4.3) and OTU-1013670 *Halomonas* (5.6)
Rhizosphere	New 0 CleanUp Reference OTU-2374 Hyphomonadaceae (2.1); OTU-1101451 Micrococcus (2.2); New 0 CleanUp Reference OTU-6111 Chloroflexi (2.2); New 0 CleanUp Reference OTU-7251 Caldilineaceae (2.3); **OTU-347529 *Turicibacter*** (2.4); OTU-285497 Caulobacteraceae (2.8); New 0 CleanUp Reference OTU-1351 SC-I-84 (3.2); New 0 CleanUp Reference OTU-7203 Aerococcaceae (3.6); OTU-1110303 Sinobacteraceae (3.6); OTU-1082294 Streptococcus (5.9)

[a] Value between parenthesis is the ratio of the relative abundance of the OTU in the biofertilizer amended soil versus the relative abundance of the OTU the unamended soil, [b] The relative abundance of the OTUs in bold font increased in more than one soil.

Supplementary Table S8. Effect of time (44, 89 and 130 days) on bacterial groups in the uncultivated, non-rhizosphere and rhizosphere soil left unamended or applied with biofertilizer or sterile biofertilizer using a compositional approach, i.e. analysis of differential abundance taking sample variation into account (ALDEx2 package, Gloor et al.[2]).

	Unamended	Biofertilizer	Sterile biofertilizer
		Effect of time on bacterial groups	
Uncultivated	*Citromicrobium* (0.049) [a], *Haliangium* (0.035), *Kaistobacter* (0.027), *Plesiocystis* (0.042)	*Acinetobacter johnsonii* (0.038), *Delftia* (0.038), *Kaistobacter* (0.041)	*Citrobacter* (0.042)
Non rhizosphere	Firmicutes (0.044)<br>*Citrobacter* (0.042), *Halomonas* (0.039), *Lactobacillus zeae* (0.044), *Lactococcus* (0.047), *Pseudomonas* (0.047), *Vibrio metschnikovii* (0.049)	*Halomonas* (0.039), *Sorangium cellulosum* (0.039), *Virgisporangium* (0.035)	Tenericutes (0.046)<br>*Asteroleplasma* (0.0048), *Devosia* (0.039), *Kaistobacter* (0.033), *Sphingomonas* (0.043), *Stenotrophomonas* (0.042)
Rhizosphere	Bacteroidetes (0.044), Unassigned bacteria (0.037)<br>*Aeromicrobium* (0.049), *Flavisolibacter* (0.046), *Geobacter* (0.049), *Halomonas* (0.046), *Kaistobacter* (0.027), *Paenibacillus* (0.036), *Pseudomonas* (0.045), *Stenotrophomonas* (0.034)		*Acinetobacter* (0.027), *Burkholderia* (0.043), *Chryseobacterium* (0.039), *Dyadobacter* (0.040), *Halomonas* (0.027), *Kaistobacter* (0.036), *Parvibaculum* (0.028), *Sorangium cellulosum* (0.035), *Stenotrophomonas* (0.035), *Virgisporangium* (0.042)

[a] Value between parenthesis: the expected values of the Kruskal-Wallis test for each feature obtained with aldex.kw argument with converted sequence data using the centred log-ratio transform test returned by the aldex.clr argument (ALDEx2 package).

References to the Supplementary Material

1. Mair, P. & Wilcox, R. Package 'WRS2' Type Package Title A Collection of Robust Statistical Methods. (2019). Available at: https://r-forge.r-project.org/projects/psychor/.
2. Gloor, G. *et al.* Analysis of differential abundance taking Sample Variation Into Account: Package 'ALDEx2'. **1.18.0**, 5 (2019).
